# Psychological well-being in midlife following early childhood intervention

**DOI:** 10.1017/S0954579421001528

**Published:** 2022-01-24

**Authors:** Christina F. Mondi, Arthur J. Reynolds

**Affiliations:** 1 Institute of Child Development, University of Minnesota, Twin Cities, Minneapolis, USA; 2 Brazelton Touchpoints Center, Division of Developmental Medicine, Boston Children’s Hospital, Boston, USA; 3 Harvard Medical School, Boston, USA

**Keywords:** early childhood education, mechanisms of early intervention, preschool, psychological wellbeing, poverty

## Abstract

The present study is the first to examine the relations between participation in a public early childhood intervention (the Child-Parent Center (CPC) program) and psychological well-being (or, positive functioning) into early mid-life. Data are drawn from the Chicago Longitudinal Study (CLS), which has followed a cohort of 1,539 individuals who grew up in urban poverty for over four decades. Approximately two-thirds of the original study cohort participated in the CPC program in early childhood; the rest comprise a demographically matched comparison group. Participantsâ€™ psychological functioning at age 35–37 was assessed using the Ryff Scales of Psychological Wellbeing. Results support a positive relationship between CPC preschool participation and long-term psychological wellbeing. Moderated mediation (e.g., whether CPC effects on wellbeing differ across subgroups) and potential mechanisms across multiple social-ecological levels (according to the 5-Hypothesis Model of early intervention) are also empirically investigated. Future directions for child development research, early childhood intervention, and public policy are discussed.

The U.S. Department of Health and Human Services has identified improvement of mental health as a Healthy People 2030 objective, and emphasized that achieving this will require addressing social determinants of health (e.g., social and community context, education access and quality) (Office of Disease Prevention and Health Promotion, n.d.). This call to action acknowledges the systemic health disparities which have persisted for centuries in the U.S., and which are often evident starting in the first years of life. For example, in 2019, over 10 million U.S. children and adolescents lived in poverty, including a disproportionate number of Black, Hispanic, and American Indian/Alaska Native children (Semega et al., 2020). Poverty, in turn, has been linked to increased risk of negative health outcomes across the lifespan – including increased prevalence rates of mood and anxiety disorders, substance misuse, and suicide (e.g., Barreto & McManus, 1997; Koblinsky et al., 2006; Sareen et al., 2011; Saulsberry et al., 2013).

The latter findings underscore a critical need for prevention and intervention initiatives which will reduce socioeconomic and racial/ethnic disparities in mental health. However, is the prevention of psychopathology a sufficient goal? Emory Cowen, a pioneer in the field of prevention science, argued that primary prevention serves dual purposes: (a) preventing the development of serious psychopathology and (b) promoting positive psychological functioning, or *psychological well-being* (PW; Cowen, 1973, 1977; Cowen & Durlak, 2000). From this perspective, initiatives should aim not only to reduce rates of psychopathology, but to enhance skills and protective factors which will enable individuals to withstand adversity and lead meaningful, productive lives (e.g., Galderisi et al., 2015; Halle & Darling-Churchill, 2016; Keyes, 2002; Masten & Curtis, 2000). To this end, the present study investigates whether participation in an early childhood education program is related to long-term PW (through early midlife) in a low-income, predominately African American cohort.

Part I of this Introduction will discuss the construct and measurement of PW, group differences in PW, and the potential role of early childhood intervention in supporting PW. Part II will discuss mechanisms by which early childhood intervention may promote long-term PW.

## Present study part I: Early childhood intervention and later PW

### PW

Mental health can be conceptualized on a continuum ranging from psychopathology to PW (also known as positive psychological functioning or flourishing) (Cowen, 1973, 1977; Cowen & Durlak, 2000; Darling-Churchill & Lippman, 2016; Diener et al., 1999; Keyes, 2002). PW is not the mere absence of psychopathology symptoms; rather, individuals on this end of the continuum possess psychological skills and resources that enable them to withstand adversity and build meaningful lives (Camfield & Skevington, 2008; Diener, 1984; Diener & Diener, 1995; Ryff & Keyes, 1995; Ryff & Singer, 1996; World Health Organization, 2014).

Ryff (1989a) drew on empirical research from numerous disciplines and theories (e.g., lifespan development, clinical theories of personal growth, positive mental health) to develop an integrated model of personal development. This model is comprised of six theoretically derived dimensions of PW, including (a) positive appraisals of oneself and one’s past life (Self-Acceptance); (b) the capacity to manage effectively one’s life and environment (Environmental Mastery); (c) the presence of high-quality interpersonal ties (Positive Relations with Others); (d) the belief that one’s life is purposeful and meaningful (Purpose in Life); (e) a sense of continued growth and development as an individual (Personal Growth); and (f) a sense of self-determination (Autonomy) (Ryff, 1989a, 1989b; Ryff & Singer, 2006; Schmutte & Ryff, 1997, p. 551). Ryff and colleagues translated these constructs into a now widely used assessment scale, the Ryff Scales of Psychological Well-being (RSPWB; Ryff, 1989b; Ryff & Keyes, 1995). Ryff and Singer (1996, 2006) have extensively described the process by which the RSPWB were developed and summarized several decades of evidence supporting their multidimensionality.

### PW across groups

Researchers have also investigated similarities and differences in PW across various groups. For example, several studies have indicated that adverse childhood experiences (ACEs) and poverty predict lower levels of PW in adulthood – relationships which are likely related to complex alterations in social support, neurobiological development, and more following early adversity (e.g., Herrenkohl et al., 2012; Huang et al., 2021; Kong, 2018; Mosley-Johnson et al., 2019). Meanwhile, findings regarding PW and gender have been more mixed, with Matud et al. (2019) noting that while “literature has shown differences between men and women in some psychological well-being dimensions, such differences generally vary depending on other factors such as age, culture, or roles played” (p. 3530).

Building on Matud et al. (2019) comments, previous work has also explored PW patterns across racial and ethnic groups. In the United States, Black, Indigenous, and other communities of color face systemic racism in numerous areas of daily life (e.g., the educational, healthcare, and criminal justice systems), which contributes in myriad ways to persistent health and economic disparities (e.g., Roberts & Rizzo, 2021; Yearby, 2020). To this end, various studies have linked racism-related stressors to lower PW and higher levels of mental health problems (e.g., Buchanan & Fitzgerald, 2008; Paradies et al., 2015; Pieterse & Carter, 2007).

It is also important to underscore the many assets and strengths of Black, Indigenous, and other communities of color – for example, high levels of extended kin, neighborhood, and spiritual support – which may promote PW, even in contexts of adversity (Hunn & Craig, 2009; Littlejohn-Blake & Darling, 1993; Ward & Mengesha, 2013). In a notable study, Ryff et al. (2004) reported on the use of the RSPWB in the longitudinal Mid-life in the United States (MIDUS) study, specifically comparing White Americans (nationally), African Americans (both nationally and in New York City specifically), and Mexican Americans (in Chicago). They reported overall similar subscale- and age-related RSPWB patterns across racial and ethnic groups, comparable to what had been found in previous community-based studies. However, stronger gender differences were found than in previous studies, with evidence for PW disadvantages among females of color. They also reported that, across subgroups, being a person of color was associated with higher PW, even after controlling for factors like education and perceived discrimination. They commented:

These findings … draw attention to a frequently neglected phenomenon, namely, that the presence of the negative in the lives of oppressed groups does not automatically imply an absence of the positive. That is, advantage in well-being may sometimes exist concomitantly with negative outcomes … [and] certain aspects of well-being, such as having a high sense of self-regard, mastery, and personal growth, may actually be honed by challenge, applied in this case to the difficulties of minority life (p. 418).

These remarks and findings underscore the importance of moving beyond measurement of correlations between race and ethnicity, poverty, and PW, toward assessment of the unique social-ecological contexts, risk and protective factors which affect different groups. Such investigations may inform intervention efforts to support lifelong PW and resilience.

### PW and early childhood intervention

In recent decades, researchers, practitioners, and policymakers have increasingly advocated for the implementation of interventions that will promote PW starting in the first years of life. In the United States, large-scale early care and education (ECE) interventions rose to prominence during the “War on Poverty” of the 1960s. Project Head Start was initially administered through the newly established federal Office of Economic Opportunity, and the Child-Parent Center (CPC) preschool program was established shortly thereafter with funding from Title I of the Elementary and Secondary Education Act of 1965. The initial aims of most ECE interventions were to promote academic achievement and educational attainment, though many also had complementary goals of enhancing children’s socioemotional functioning (e.g., self-esteem, motivation, social competence) (Raver & Zigler, 1997; Zigler, 1970, 1973). Nonetheless, at the public policy level, the continued funding of ECE programs has historically been predicated on academic, occupational, and related outcomes. Longitudinal evaluations of public ECE programs have thus focused much more on the latter domains, and less so on program impacts on broader well-being. As such, important questions remain about whether ECE intervention can reduce not only educational disparities, but also disparities in health and PW.

To our knowledge, the present study is the first to evaluate the relationship between participation in a public ECE intervention and long-term PW (into early midlife). Data are drawn from the Chicago Longitudinal Study (CLS), which has followed a cohort of 1,539 individuals who grew up in urban poverty for over four decades. Approximately two-thirds of the original study cohort participated in the CPC program (a school-based intervention, described further in the Method) in early childhood. Part I of this study investigates two questions:

*Question 1: “Is CPC program participation (beginning in preschool) associated with greater PW in early midlife (relative to a matched comparison group)?”.* This question examines the main effect of CPC program participation on long-term PW. It is hypothesized that CPC graduates will exhibit significantly higher levels of PW relative to a comparison group in early midlife (age 35–37).

*Question 2: “Does the relationship between CPC program participation and early midlife PW differ for key subgroups (based on sex, early ACE history, and early family sociodemographic risk)?”.* This question examines whether the main effect of CPC participation on PW is moderated/modified for different subgroups. The present study will examine whether CPC intervention exerts different effects on three subgroups of low-income individuals: (a) males (vs. females); (b) individuals who experienced high levels of family-level sociodemographic risk between ages 0 and 3 years (vs. those with relatively lower levels of risk); and (c) individuals who experienced ACEs between ages 0 and 5 years (vs. those who did not). Consistent with research indicating that males and participants with the highest cumulative risk benefit the most from intervention (e.g., Magnuson et al., 2016; Ou & Reynolds, 2010; Reynolds et al., 2011), it is hypothesized that CPC intervention will exert greater psychological benefits for males, individuals affected by high levels of early sociodemographic risk, and ACE-affected individuals than for the overall sample.

## Present study part II: Investigation of mechanisms

Part II of the present study investigates the potential mechanisms by which participation in an early childhood education program (CPC) may promote long-term PW. This work is guided by the Five-Hypothesis Model (5HM), which provides a transactional-ecological framework for understanding the effects of ECE interventions (Reynolds, 2000). The 5HM posits that five primary mechanisms underlie ECE intervention effects (e.g., Reynolds, 2000; Reynolds et al., 2004; Reynolds et al., 2019). These mechanisms closely parallel ones that have been investigated in other ECE studies (e.g., Campbell & Ramey, 1995; Schweinhart et al., 1993) and are briefly described below.

### The 5HM

#### Cognitive-scholastic advantage

This mechanism posits that children who participate in ECE programs exhibit improved cognitive and scholastic abilities upon school entry (as measured by standardized achievement tests, language, and literacy skills). This “cognitive advantage”, in turn, is hypothesized to initiate positive trajectories of academic performance (e.g., better grades, reduced rates of grade retention, higher rates of high school completion), which may subsequently lead to improvements in self-esteem, motivation, and behavior (Schweinhart et al., 1993). Regarding mental health specifically, multiple studies have demonstrated that cognitive abilities and academic achievement predict both short- and long-term mental health (e.g., Chesmore et al., 2016; Huang, 2015; McCarty, 2008; Melkevik et al., 2016; Robles-Piña et al., 2008).

#### Family support behavior

This mechanism posits that ECE promotes children’s long-term adjustment by enhancing the expectations, caregiving behaviors, and overall well-being of their primary caregivers. Many ECE programs have historically included parent education programs (e.g., workshops on child development, GED completion courses) and ample opportunities for parent involvement in and out of the classroom. In regard to mental health specifically, a robust body of research has demonstrated the importance of family support and parent involvement for children’s mental health outcomes (e.g., Suldo et al., 2012; Stice et al., 2004). Parents who are empowered and supported by a close-knit school community are less likely to experience mental health problems themselves, are less likely to engage in negative parenting behaviors, and are better able to promote their children’s socio-emotional development (Green et al., 2014; Mersky et al., 2011). On the contrary, parents and families who exhibit low levels of support and involvement may confer psychological risk to their children, exacerbating their long-term risk for mental health problems (e.g., Sagrestano et al., 2003).

#### School and community support

This mechanism posits that the effects of participation in high-quality ECE programs are likely to be maintained if graduates subsequently attend high-quality school-age programs. Previous research has indicated that Head Start graduates are more likely to attend lower quality elementary schools than their non-Head Start peers, and that the effect of Head Start on long-term academic achievement was a function of the achievement levels of the elementary schools that children attended (Currie & Thomas, 2000). Regarding mental health specifically, previous research has shown that children who have positive perceptions of their school’s climate and social connectedness exhibit better long-term mental health (e.g., Bond et al., 2007; Suldo et al., 2012). It is also important to note that whereas many ECE programs end after preschool or Kindergarten (including Head Start), others continue through early elementary school. For example, the CPC program can run from preschool through third grade, depending on the school site. Research has revealed a dose-response relationship between the length of CPC participation and a variety of outcomes (Reynolds et al., 2011). This relationship is thought to be driven by reductions in school mobility, the latter of which disrupts children’s learning and social relationships (e.g., Boon, 2011; Simpson & Fowler, 1994).

#### Motivational advantage

This mechanism posits that high-quality ECE programs promote long-term well-being by enhancing children’s motivation and self-concept (e.g., task persistence, perceived competence, and self-efficacy) in early childhood. This “motivational advantage”, in turn, may act as a promotive or protective factor as children continue through school and confront various stressors and adversities. As previously discussed, little research has examined the relationship between ECE intervention, self-esteem, and self-concept; the few extant studies have found little evidence to support significant beneficial effects (see review in D’Onise et al., 2014). Other papers, however, have suggested that ECE intervention may increase children’s perceived scholastic competence and expectations for their future (Campbell et al., 2002; Mondi et al., 2017), which may meaningfully contribute to long-term socio-emotional adjustment. As such, this hypothesis merits additional investigation.

#### Socioemotional adjustment

This mechanism posits that high-quality ECE programs promote long-term well-being by enhancing children’s socio-emotional skills (e.g., interpersonal skills, self-regulation abilities, internalization of social rules) in early childhood. As has been discussed throughout this paper, early SEL provides a critical foundation for lifelong psychological well-being, above and beyond cognitive and other factors.

#### Interplay across mechanisms

The 5HM is designed as an integrative system, in which each mediator makes significant and complementary contributions to participant outcomes (Reynolds & Ou, 2011). In many cases, the hypotheses may reinforce and interplay with each other (e.g., high levels of motivation may enhance academic engagement, leading to greater Cognitive-Scholastic Advantage and subsequently even higher levels of motivation); in others, one or two hypotheses may make greater proportional contributions to a particular outcome. As such, it is valuable to investigate both the separate and cumulative contributions of each hypothesis, with attention to the model that is the best statistical and theoretical fit (Reynolds & Ou, 2011; Reynolds et al., 2004).

Many studies have investigated the contributions of one or more of the 5HM mechanisms on well-being, but relatively few have comprehensively examined the complete model. In a follow-up study of 1,404 CPC graduates, Reynolds and colleagues (2004) reported that a LISREL model including variables of School and Community Support, Cognitive-Scholastic Advantage, and Family Support Behavior accounted for 58% of the effect of CPC preschool participation on high school completion, and 79% of the direct effect of CPC preschool on juvenile arrest. LISREL analyses in a subsequent paper indicated that the complete 5HM model accounted for 79% of the direct effect of CPC preschool on depressive symptoms in emerging adulthood. A major implication of these results is that consistent with the developmental psychopathology perspective, it is the complex interplay of mechanisms over time that shapes long-term outcomes, rather than any one mechanism in isolation. Furthermore, the respective mechanisms are likely to exert differential impacts on children who are affected by unique risk and protective factors.

**Figure 1.**
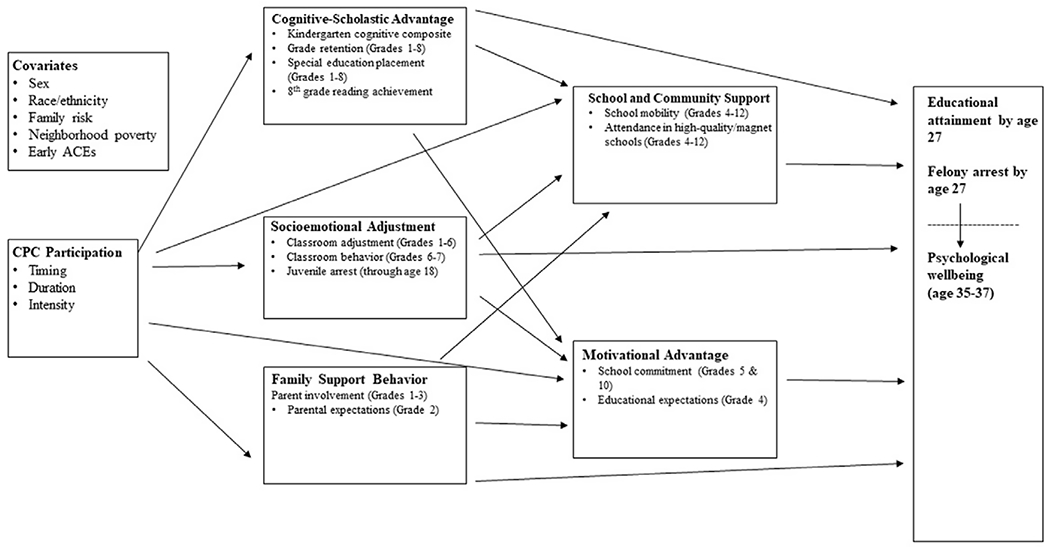
Model of CPC effects on midlife PW.

Drawing on this background, Part II of the present study explores two questions:

*Question 3: “Does socioemotional adjustment mediate the relationship between CPC preschool participation and long-term PW?”* This question examines the extent to which 5HM mediators account for the overall main effect of program participation on PW in early midlife (Reynolds, 2000). Previous research outside the ECE field has uncovered an inverse relationship between childhood socioemotional skills and later mental health problems (Jones et al., 2015). As such, it is hypothesized that Socioemotional Adjustment will mediate the relations between CPC participation and long-term PW. It is predicted that the other 5HM mediators (Cognitive-Scholastic Advantage, Motivational Advantage, Family Support Behavior, and School and Community Support) will also mediate the latter relationship, but to a lesser degree (Figure 1).

*Question 4: “Do mediational pathways from CPC preschool participation to early midlife PW vary by participant subgroup (based on sex, early sociodemographic risk, and early ACE history)?”* This question investigates whether there is moderated mediation for the three categories of participant subgroups described in Question 2. It is hypothesized that Socioemotional Adjustment will more strongly mediate the relations between CPC participation and long-term PW for males, individuals with high levels of sociodemographic risk, and individuals with early ACE histories.

## Method

### Sample and design

The present study examines the long-term effects of the CPC program on PW. The CPC program’s founders believed that a whole-child instructional approach, coupled with intensive family support services, would bolster school readiness and academic achievement (Graue et al., 2004; Sullivan, 1971). Drawing on the emerging science of child development, they designed a preschool to third grade (P-3) program that emphasized five key elements: (a) early educational enrichment beginning no later than age four; (b) a structured instructional approach that emphasized language and literacy; (c) a menu-based parent involvement and education program; (d) provision of and referrals to health and social services; and (e) continuity between preschool and third grade (Reynolds et al., 2003). The implementation of these program elements was overseen by a core leadership team at each site. This team included the school principal, a head teacher, a parent-resource teacher, and a school-community representative (Reynolds, 2000; Sullivan, 1971). Whenever feasible, the program’s P-3 services were colocated in the same building; when this was not possible, preschool services were offered in centers in close proximity to elementary school buildings, or at community sites further away from the schools into which students ultimately matriculated (Reynolds & Mondi, 2016).

The present study draws on data from the CLS, a quasi-experimental investigation of the CPC program’s long-term effects. Since 1985, the CLS has tracked the development of a cohort of 1,539 individuals who were born in 1979 or 1980 and who grew up in contexts of urban poverty (Reynolds, 2000). The original study sample was 92.9% African American and 7.1% Hispanic. Intervention group members (*N* = 989) attended the CPC pre-school program at twenty different sites when they were 3 or 4 years old. CLS comparison group members (*N* = 550) did not attend CPC preschool, but participated in full-day public kindergarten (the usual early childhood intervention available in their neighborhoods) at randomly selected Chicago sites with similar poverty characteristics as the CPC sites. According to the study’s Principal Investigator, “children in the comparison group of this quasi-experimental study did not enroll in the CPCs primarily because they did not live in the neighborhood of a center. Thus, geographic location rather than family motivation determined nonparticipation” (Reynolds, 1997, p. 9). Previously published research has confirmed that the intervention and comparison groups had comparable baseline characteristics (e.g., age, intervention participation, neighborhood poverty) (Reynolds & Ou, 2011; Reynolds et al., 2011).

### Procedure

The present study’s independent variables are drawn from CLS data collected over more than three decades. Teacher surveys were conducted annually between kindergarten through sixth grades. Parent surveys were conducted at five time-points, when participants were in second, fourth through sixth, and eleventh grades. Participant surveys were conducted annually from third through sixth grades and at two time-points in high school. Researchers also collected school and government records at various time-points.

Three surveys have been administered since participants reached adulthood (at ages 20–21, 22–24, and 35–37, respectively). CLS researchers have also periodically collected administrative and government records of college attendance, criminal justice system involvement, income, and public aid.

The present study’s PW outcome data are drawn from the follow-up survey which was conducted from 2012 to 2017, when participates were ages 35–37. The survey asked about participants’ past and present functioning in multiple domains (e.g., educational, occupational, health, family life). 1,107 participants (71.8% of the original CLS sample and 76.7% of the living sample) completed the survey by phone, by mail, or in-person. Table 1 displays demographic characteristics of the original CLS sample versus the subsample that was recovered in the current follow-up.

### Measures

The present study utilizes a variety of report sources (e.g., participant, parent and teacher reports; administrative records) to minimize potential reporting bias. Alternative covariate specifications (e.g., continuous, dichotomous, and threshold variables) were also examined to determine the stability and consistency of observed patterns.

#### Age 35–37 outcomes

Information about PW at age 35–37 was drawn from self-reports on the Ryff Scales of Psychological Well-being (RSPWB; Abbott et al., 2006; Ryff & Keyes, 1995) (*N* = 1,105–1,117). Participants rated their agreement with 18 items (e.g., “For me, life has been a continuous process of learning, changing, and growth; I have confidence in my own opinions, even if they are different from the way most other people think”) on a 5-point Likert scale, ranging from “1” (“*Agree strongly*”) to “5” (“*Disagree strongly*”).

Consistent with previous research, the present study analyzed the sum of participants’ 18 item ratings on the RSPWB, yielding a total PW score. Scale reliabilities for the six original subscales identified by Ryff and Keyes (1995) were low; as such, these were not outcomes of focus in the present study.

#### Part I: Early childhood intervention and later PW

Part I of this study (Questions 1 and 2) examines the following predictors of PW:

#### CPC intervention

The present study included two measures of CPC participation: (a) a dichotomous variable indicated any CPC preschool participation and (b) a dichotomous variable indicating any participation in the CPC follow-on program between first and third grade.

#### Sex

A dichotomous variable was created to indicate whether participants were male (1) or female (0). This information was drawn from school records.

#### Race/ethnicity

A dichotomous variable was created to indicate whether participants were Black (1) or not Black (0). This information was drawn from school records.

#### Low birthweight

A dichotomous variable was created to indicate whether participants were of low birthweight (<2500 g), based on birth certificate records.

#### Neighborhood poverty

This variable indicates the percentage of individuals in the participant’s birth census tract that had incomes under the federal poverty line in 1980 (the year that most participants were born). This information was drawn from government records.

#### Early ACEs (ages zero to five)

ACE information was drawn from self-reports and administrative records. Participants completed a measure resembling the ACE checklist from the Kaiser Permanente ACE Study during the age 22–24 survey (Felitti et al., 1998). This measure assessed whether participants had experienced six categories of ACEs: (a) prolonged absence or divorce of parents; (b) death of a parent, sibling, or close friend; (c) frequent family conflict; (d) parental substance abuse; (e) witnessing a violent crime; and (f) being a victim of a violent crime. Participants also reported their age at the time of each ACE (ages zero to five, six to ten, ten to fifteen, or sixteen and up, respectively). Participants were not asked to report on childhood maltreatment due to the sensitivity of the topic and the likelihood of underreporting (Edwards et al., 2003). Instead, physical abuse, sexual abuse, and neglect were assessed using court and county records from the Department of Child and Family Services.

Participants completed the same ACE measure during the Age 35–37 Survey. These ACE data were utilized for participants who did not complete the age 22–24 survey. Among participants who completed both surveys, data from the Age 35–37 Survey were used to supplement the age 22–24 data if the participant reported new information about ACEs at age 35–37. The present study focused on early ACEs occurring between ages zero and five. Cumulative early ACE scores were calculated by summing the number of ACEs that reportedly occurred during this period.

#### Early sociodemographic risk (ages zero to three)

Participants’ early sociodemographic risk between ages zero and three was assessed using a continuous risk index. This index was computed by summing eight risk factors that were dichotomously coded for presence (1) or absence (0) at any time before age three: (a) mother was under age 18 at the participant’s birth; (b) mother was not a high school graduate; (c) mother was unemployed or employed part-time; (d) participant lived in a single parent household; (e) participant lived in a household of four or more children; (f) participant lived in a school attendance area where at least 60% of households were impoverished; (g) family income was below 185% of the federal poverty level; and (h) participant was eligible for free lunch. Information was drawn from family and participant surveys, the Illinois Department of Public Health, Chicago Public School records, and the Illinois Public Assistance Research database. Approximately 10% of cases with missing data were imputed using the Expectation Maximization (EM) algorithm (Schafer, 1997).

### Subgroups

#### Sex

See description above.

#### Early sociodemographic risk

See description above. A dichotomous variable was created to indicate whether participants had four or more of the eight risk indicators.

#### Early ACEs

See description above. A dichotomous variable was created to indicate whether participants had experienced at least one ACE between ages zero and five.

### Part II: Investigation of mechanisms

Part II of this study (Questions 3 and 4) examines 5HM mechanisms as possible mediators of the relationship between CPC participation and PW. The 5HM mechanisms were measured as follows:

#### Socioemotional adjustment

Socioemotional Adjustment was assessed using four indicators: (a) teacher ratings of classroom adjustment (grades one through six); (b) participants’ self-reported perceived competence (grades three through six); (c) teacher ratings of classroom adjustment (grades six through seven); and (d) juvenile arrest status (through age eighteen) (Figure 1).

Classroom adjustment between grades one and six was assessed using annual teacher reports. Teachers rated participants’ classroom behaviors in relation to six items: (a) “concentrates on work”; (b) “follows direction”; (c) “is self-confident”; (d) “participates in group discussion”; (e) “gets along well with others”; and (f) “takes responsibility for actions”. Ratings were made on a 5-point Likert scale, ranging from “1” (“*poor/not at all*”) to “5” (“*excellent/very much*”). The present study examined the *mean* of participants’ ratings across grades. Missing scores (3.5% of the sample, due to teacher non-response) were *mean* score imputed. The reliabilities of the scales ranged from .89 to .93 across grades.

Perceived competence was assessed using annual self-reports from grades three through six. During these grades, participants rated their agreement with 10 to 12 items (e.g., “I am smart”; “My classmates like me”) on four-point Likert scales, ranging from “1” (“*strongly disagree*”) to “4” (“*strongly agree*”). Despite slight differences in item content across years, the annual scales were significantly correlated with each other (*r* = 0.30). Missing scores (6.8% of the sample) were *mean*-imputed, and *mean z*-scores were used in analyses. The reliabilities of the scales ranged from 0.69 to 0.76 across grades.

Classroom adjustment in grades six and seven was assessed using annual teacher reports. Teachers rated participants’ classroom adjustment using the Teacher-Child Rating Scale (T-CRS; Hightower et al., 1986). Teachers rated how well various statements (e.g., “is friendly towards peers”, “works well without supervision”, “copes well with failure”) described participants on a five-point Likert scale, ranging from “1” (“*not at all*”) to “5” (“*very well*”). Items yield seven subscales: (a) Frustration Tolerance; (b) Assertive Social Skills; (c) Task Orientation; (d) Peer Social Skills; (e) Acting-Out Behavior; (f) Shy-Anxious Behavior; and (g) Problem Behaviors/Learning Problems. The present study examined the average of participants’ ratings on each subscale between sixth and seventh grade. Missing scores were *mean*-imputed. The reliabilities of the subscales ranged from 0.79 to 0.96 across grades.

Juvenile arrest information was drawn from court and county records. Record searches for formal petitions for criminal charges were conducted at the Cook County Juvenile Court and two additional Midwest locations. Searches were only conducted for participants who resided in Chicago at age 10 or older. Searches were conducted without knowledge of individuals’ intervention statuses, were repeated twice for 5% random samples, and were compared against computer records. The present study utilized a dichotomous variable indicating whether participants were ever arrested between the ages of 10 and 18.

#### Cognitive-scholastic advantage

Cognitive-Scholastic Advantage was assessed using four indicators: (a) Kindergarten cognitive abilities; (b) grade retention (grades one through eight); (c) special education placement (grades one through eight); and (d) eighth grade reading scores (Figure 1).

Information about cognitive abilities at Kindergarten entry (age six) was drawn from the Iowa Test of Basic Skills (ITBS Level 5; Hieronymus et al., 1982), which was administered in October of the kindergarten year. The ITBS assesses developed cognitive abilities, and includes assessments of listening and word analyses, vocabulary, and comprehension of mathematical concepts. The test was normed in 1978, and internal consistency reliability has been reported at .94 (Hieronymus et al., 1982). The present study analyzed participants’ composite developmental standard scores.

Grade retention was assessed using information from school records. A dichotomous variable was created to indicate whether participants were ever retained between grades one and eight (ages seven through 14) was created.

Special education placement was assessed using information from school records. A dichotomous variable was created to indicate whether participants ever received special education services between grades one and eight (ages seven through 14).

Information about reading abilities in eighth grade (age 14) was drawn from the Iowa Test of Basic Skills battery (ITBS; Level 14 or 13; Hieronymus & Hoover, 1990), which was group-administered in April of participants’ eight-grade year. The present study examined scores from the reading comprehension subtest, which consisted of 58 items. The test was normed in 1988, and the reliability of the scale was .92. Data were missing for 9.9% of cases. The scores of 6.7% of the latter cases were estimated using ITBS scores from previous grades. Data were imputed using the EM algorithm for another 3.2% of missing cases that were also missing scores from other grades.

#### Family support behavior

Family Support Behavior was assessed using two primary indicators: (a) parent involvement in children’s education (grades one through three) and (b) parental expectations for children’s educational attainment (grade two) (Figure 1).

Parent involvement was assessed using annual teacher reports from grades one through three. Teachers annually rated parents’ participation in school activities using a five-point Likert scale, ranging from “1” (“*poor*”) to “5” (“*excellent*”). The present study examined the *mean* of teachers’ ratings across grades. Missing data were imputed using socio-demographic measures.

Parental expectations for children’s educational attainment were assessed using parent reports from grade two. At this time-point, parents rated their expectations for their children’s educational attainment on a five-point Likert scale, ranging from “1” (“*some high school*”) to “5” (*graduate school*). Previous research has demonstrated that these measures exhibit adequate reliability and validity (Miedel & Reynolds, 1999).

#### School and community support

School and Community Support was assessed using two primary indicators: (a) school mobility (grades four through 12) and (b) attendance in high-quality schools (grades four through 12) (Figure 1).

School mobility was assessed using information from school records. A continuous variable was created to indicate the number of times that participants changed schools between grades four and 12.

Attendance in high-school schools was assessed using information from school records. A dichotomous variable was created to indicate whether participants ever attended high quality or magnet schools between grades four and 12, including (a) citywide magnet schools, which employed selective enrollment policies and required good academic performance or (b) schools in which 40% or more of the student body performed at or above national averages on ITBS reading and math tests.

#### Motivational advantage

Motivational Advantage was assessed using two primary indicators: (a) school commitment (grades five and ten) and (b) educational expectations (grade four) (Figure 1).

School commitment was assessed via annual self-reports in grades five and six. Participants rated their agreement with thirteen items (e.g., “I try hard in school”, “School is important”, “I get bored in school”) on a three-point Likert scale, with higher scores indicating higher commitment. Items differed slightly across grades; as such, the present study calculated participants’ *z*-scores and examined the average of participants’ *z*-scores across grades.

Educational expectations were assessed using self-reports from grades four and ten. At these time-points, participants responded to the question “How far in school do you think you will get?” on a five-point scale, ranging from 1 (grade eight) to 7 (doctorate degree). Consistent with previous research, a dichotomous variable indicating whether participants expected to attend college was utilized (Ou & Reynolds, 2008). Participants’ responses at age ten will be used as the main measure unless they were missing data at that time point, in which case their age sixteen responses will be used.

#### Educational attainment

Educational attainment was assessed using aggregate data obtained from administrative records from state college, K-12 schools, and participant surveys. An ordinal variable was created to indicate the highest grade that participants had completed by age 28 (ranging from seven to 16, with individuals who passed the GED assigned a value of 12).

#### Criminal justice system involvement in adulthood

Criminal justice system involvement was assessed using aggregate data from administrative court and county records, Department of Motor Vehicle Records, and CLS participant surveys. A dichotomous variable was created to indicate whether participants had ever been arrested for a felony as of age 35–37.

### Data analysis plan

#### Attrition correction

Participant attrition over time is a major challenge for longitudinal studies. Systematic attrition may affect the inference and generalizability of study findings, especially if the reasons for attrition (e.g., poor mental health, social isolation) are meaningfully related to study variables. As such, the present study utilized inverse probability weighting (IPW) methods to reduce potential bias from differential attrition, in keeping with recent CLS papers (e.g., Mondi et al., 2020; Reynolds et al., 2011). IPW employs all available data to estimate complex adjustments, independent of the outcome specification model (Mondi et al., 2020; Seaman & White, 2013). IPW has been shown to yield lower variances and standard errors compared to other methods (e.g., regression-based corrections, two-stage sample selection bias approaches, multiple imputation of missing data), particularly in large study samples (Hirano et al., 2003; Imbens & Wooldridge, 2009; Kurth et al., 2006; Mondi et al., 2020).

The first step of IPW is conditioning a logistic regression on a set of predictors (X) that are hypothesized to influence participants’ probabilities of sample retention (no attrition) at follow-up. This regression yields predicted probabilities of sample retention (*R* = 1). Importantly, these IPW methods assume that included participants had a greater than 0% chance of being in the study sample. As such, participants who were deceased by 2012 (when data collection for the Age 35–37 survey began) were not included in the above analyses, because they had a 0% chance of participating. Thus, the IPW methods described above yielded predicted probabilities of living participants being successfully recruited to participate.

Model fit was determined by examining whether the program and comparison groups were balanced on covariates after IPW (Li et al., 2018). The estimated CPC program effect is represented by the between-group differences in predicted probabilities for these sample retention probabilities, with two-tailed *p*-values < 0.05 indicating statistical significance.

Given the demonstrated equivalence of the CLS treatment and control groups at baseline (Reynolds, 2000) and that results did not significantly change when weighting by both program selection and attrition probabilities, the final IPW model only incorporated attrition weights. Weights were calculated separately for males and females, given previous research demonstrating differential attrition patterns by sex in the CLS (Mondi et al., 2020). Consistent with previous CLS research (Mondi et al., 2020; Reynolds et al., 2011), a comprehensive set of variables was included in the model predicting participants’ probability of being in the age 35–37 follow-up sample (see Table 2 for complete list). Table 1 displays the characteristi+cs of the Age 35–37 survey and attrition samples before and after IPW correction.

### Part I: Early childhood intervention and later PW (questions 1 and 2)

Linear regression analyses were conducted in SPSS, yielding regression coefficients and confidence intervals (IBM SPSS Statistics, IBM Corporation, Armonk, NY). Consistent with previous research, sex, race, birthweight, neighborhood poverty, early sociodemographic risk, adverse early experiences, and length of CPC participation were entered as primary moderators. For Question 2, Cohen’s *d* effect sizes were also calculated to indicate the magnitude of effects for all program comparisons, where a threshold of 0.20 is interpreted as practically significant (Cohen, 1988).

### Part II: Investigation of mechanisms (questions 3 and 4)

The present study investigated possible mediation via a difference-in-difference, or percent reduction approach (MacKinnon, 2008). This approach operationalizes mediation as the difference between the total effect of the independent variable on the dependent variable, and the direct effect of the independent variable on the dependent variable when controlling for the mediator (Fairchild & McDaniel, 2017):

In the difference-in-difference approach, the percentage of the main effect coefficient that is explained by the mediator(s) is represented by the difference between the coefficient in the main effects and mediator models. *Absent mediation* is indicated by <20% reductions in the main effect. *Partial mediation* is indicated by ~20%–40% reductions in the main effect. *Full mediation* is indicated by ~50%–100% reductions in the main effect. In cases of partial or full mediation, the addition of mediator variables to the model does not typically change the statistical significance of the main effect.

The difference-in-difference approach allows researchers to examine multiple variable paths in a single time analysis and to clearly specify the correlations among variables and the patterns of direct and indirect effects (Jeon, 2015). As such, this approach is well suited to preliminary assessments of the validity of hypothesized causal models, particularly when numerous variables are being investigated (as in the present study) (Jeon, 2015).

It is important to note the statistical assumptions of the difference-in-difference approach, including independence of residuals, non-collinearity, and lack of significant measurement error. The difference-in-difference approach can also only investigate unidirectional models; it is not possible to examine reciprocal interactions between variables (Jeon. 2015). Notably, because this is a correlational modeling approach, causation should not be inferred. The salience of the included mediators can be assessed by examining the extent to which they: (a) help to explain the main effects of the independent variable on the dependent variable (in the present study, the effect of CPC program participation on PW) and (b) uniquely contribute to the variance in the dependent variable (in the present study, PW) (Giovanell et al., 2019).

The present study followed the previously described approach, as well as the methods of previous CLS publications (e.g., Giovanelli et al., 2019). Two sets of models were estimated for PW. The first set of models assessed whether CPC program participation was associated with a statistically significant main effect on PW. If a significant main effect was discovered, the second set of models assessed the percentage of the latter effect that was explained by hypothesized mediator(s) (including each separate hypothesis, and the hypotheses in tandem with each other). 5HM mediators were entered into the full and subsample models separately and in tandem to determine whether they helped to explain the relationship between CPC program participation and later PW. Criminal justice system involvement and educational attainment variables were then entered as additional mediators, to assess the contributions of these later life experiences. For Question 4, Cohen’s *d* effect sizes were also calculated to indicate the magnitude of effects for all group comparisons, where a threshold of 0.20 is interpreted as practically significant (Cohen, 1988).

## Results

### Sample representativeness

Before presenting hypothesis-related findings, it is important to consider whether the present study sample (*N* = 1,107) is representative of the original CLS sample (*N* = 1,539). Inspection of the demographic distribution of the unweighted sample indicates that overall, the Age 35–37 Survey sample was demographically similar to the original CLS sample. This is likely attributable to the implementation of an extensive, multi-year participant tracking and interviewing process (Ou et al., 2019). The two most notable differences between the baseline and Age 35–37 Survey samples were in the domains of sex and high school completion. The Age 35–37 sample somewhat underrepresented males (49.6% in the baseline sample vs. 45.6% in the Age 35–37 sample) and participants who did not graduate from high school on-time (40.7% in the original sample versus 44.7% in the Age 35–37 sample). These are important differences, given that previous research from the CLS and other studies has shown that low-income males and participants with delayed educational attainment are at increased risk for other maladaptive outcomes, including psychopathology (Karoly et al., 2006; Mondi et al., 2017; Reynolds et al., 2011; Zigler et al., 2006). After IPW correction, the Age 35–37 Survey sample was more demographically representative of the baseline CLS sample, particularly regarding sex (49.8% male) and on-time high school graduation (38.9%). These corrections increase confidence that the present study’s findings are representative of the experiences of the original study sample.

### PW descriptive statistics

Table 3 displays the correlations among the Ryff PW items in the full, unweighted sample. Most items were significantly correlated with each other in the weak to moderate range.

Preliminary analysis of the Ryff items indicated strong reliability for the overall 18-item scale (Cronbach’s alpha = 0.75). Table 4 displays the relations among participants’ RSPWB scores and other key study variables. Significant correlations emerged between PW, CPC participation, and adult outcomes (e.g., educational attainment, criminal justice system involvement, self-reported depressive symptoms) in the expected directions.

Table 5 summarizes participants’ RSPWB scores, before and after IPW correction, in both the full sample and each subgroup. In the full weighted sample, the average total PW score (the sum of all 18 items) was 92.44 (*SD* = 11.01), with scores ranging from 52–108.

### Part I: Investigation of linkages between early childhood education and PW

*Question 1: “Is CPC program participation (beginning in preschool) associated with better PW into early midlife (relative to a matched comparison group)?”.* The text herein describes regression analyses with IPW correction. Robustness analyses with the unweighted data yielded highly similar results.

#### Full sample (Table 6)

CPC preschool participants endorsed significantly higher PW at age 35–37, compared to control group participants (*β* = 0.11, *p* < 0.01). CPC follow-on was not significantly related to later PW.

*Question 2: “Does the relationship between CPC program participation and early midlife PW differ for key subgroups (based on sex, early ACE history, and early family sociodemographic risk)?”*. The text herein describes subgroup-split regression analyses with IPW (Table 7). Robustness analyses with the unweighted data yielded highly similar results.

In sex-split analyses, CPC preschool participation was associated with higher PW in adulthood for both male (*β* = 0.12, *p* < 0.05) and female participants (*β* = 0.09, *p* < 0.05). The contributions of CPC preschool to the model were comparable across sexes (*d* = 0.05). CPC follow-on was not significantly related to later PW for males or females, making similar contributions to the model across sexes (*d* = 0.02).

In the any-ACEs group, CPC preschool was associated with significantly higher PW at age 35 in both the any-ACEs (*β* = 0.18, *p* < 0.01) and no-ACEs groups (*β* = 0.08, *p* < 0.05). CPC made significantly greater contributions to the any-ACEs model (*d* = 0.26). Participation in the elementary school CPC program was associated with lower PW in the any-ACEs group (*β* = −0.19, *p* < 0.01), but was not significantly related to PW in the no-ACEs group.

In the high early sociodemographic risk group, CPC preschool participants endorsed significantly higher levels of PW than control group members (*β* = 0.11, *p* < 0.01). CPC made similar contributions to the model across these groups (*d* = 0.03). In the lower risk group, CPC preschool participation was not significantly related to PW. CPC follow-on was not significantly related to later PW for either group.

### Part II: Investigation of mechanisms

The text herein describes the results of the weighted percent reduction analyses. Across analyses, findings were very similar across the weighted and unweighted models.

Three sets of analyses were conducted to investigate potential mediators of the relations between CPC preschool participation and PW in the full sample and in each subgroup: (a) investigations of each of the individual 5HM mediators; (b) investigation of the full set of 5HM mediators; and (c) investigation of the full set of 5HM mediators plus two key adult outcomes.

*Questions 3: “Does socio-emotional skill enhancement mediate the relationship between CPC preschool participation and long-term PW?”*.

#### Full Sample

**Figure 2.**
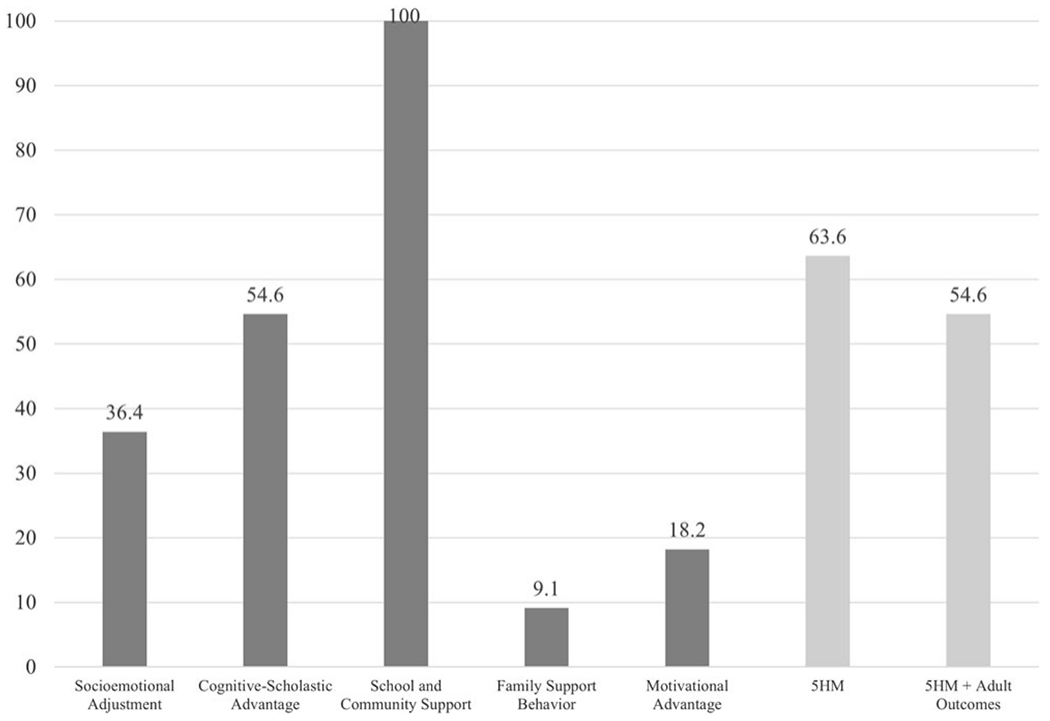
5HM mediators – Percent reductions for the effect of CPC preschool on overall PW – Full sample.

In analyses with individual mediators (Table 8), School and Community Support and Cognitive-Scholastic Advantage each fully mediated the effect of CPC preschool on PW (100.0% and 54.6% reductions, respectively). Socioemotional Adjustment partially mediated the effect of CPC preschool on PW (36.4% reduction). Motivational Advantage and Family Support Behavior were each associated with minimal reductions on the main effect of CPC preschool, indicating absent mediation (18.2% and 9.1% reductions, respectively) (Tables 8; Figure 2).

In analyses with the full set of mediators: Both the 5HM model and the 5HM-plus-adult-outcomes model fully mediated the effect of CPC preschool on PW (63.6% and 54.6% reductions, respectively) (Table 9).

*Question 4: “Do mediational pathways from CPC preschool participation to early midlife PW vary by participant subgroup?”*.

#### Sex

##### Males.

**Figure 3.**
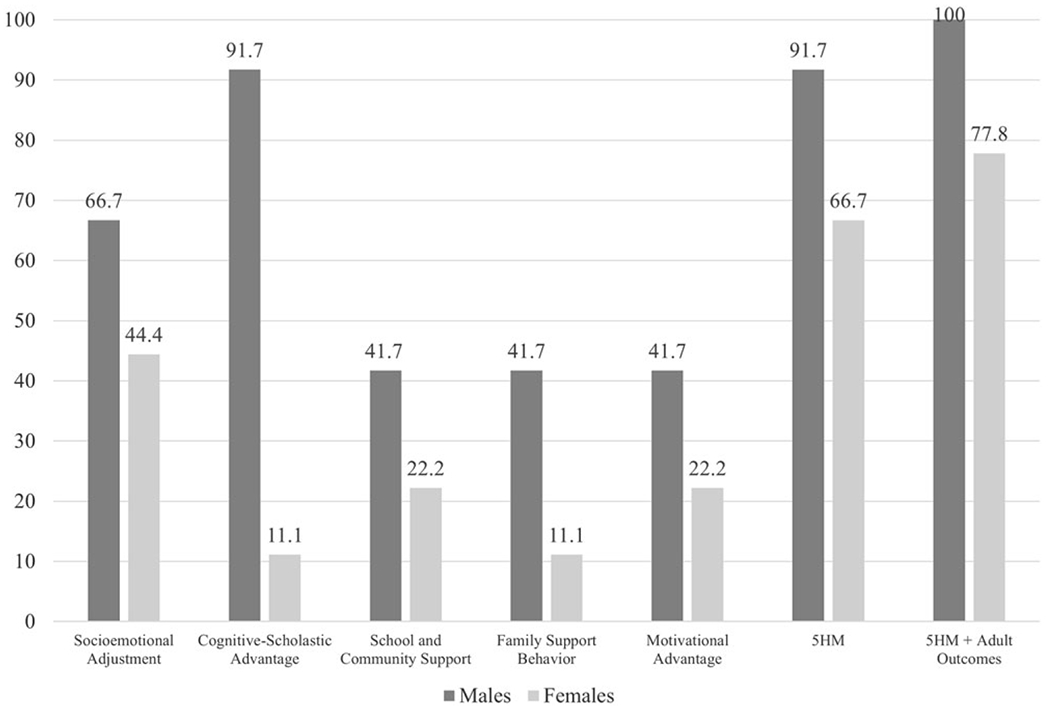
5HM mediators – Percent reductions for the effect of CPC preschool on overall PW– By sex.

In analyses with individual mediators (Table 8) Cognitive-Scholastic Advantage, Socioemotional Adjustment, Family Support Behavior, School and Community Support, and Motivational Advantage each fully mediated the effect of CPC preschool on PW (91.7%, 66.7%, 41.7%, 41.7%, and 41.7% reductions, respectively) (Tables 8 and 9; Figure 3).

In analyses with the full set of mediators (Table 9): Both the 5HM model and the 5HM-plus-adult-outcomes model fully mediated the effect of CPC preschool on PW (91.7% and 100.0% reductions, respectively).

##### Females.

In analyses with individual mediators (Table 8): Socioemotional Adjustment fully mediated the effect of CPC pre-school on PW (44.4%). School and Community Support and Motivational Advantage each partially mediated the effect of CPC preschool on PW (22.2% reduction each). Cognitive-Scholastic Advantage and Family Support Behavior were associated with minimal reductions on the main effect of CPC preschool, indicating absent mediation (11.1% reduction each).

In analyses with the full set of mediators (Table 9): Both the 5HM model and the 5HM-plus-adult-outcomes model fully mediated the effect of CPC preschool on PW (66.7% and 77.8% reductions, respectively).

##### Comparison across sexes.

CPC preschool made similar contributions across sexes in analyses with individual mediators (*d* = 0.01–0.06; Table 8), the full 5HM model (*d* = 0.04; Table 9), and the 5HM-plus-adult-outcomes model (*d* = 0.12; Table 9).

#### Early ACEs (Tables 8 and 9; Figure 4)

##### Any early ACEs.

In analyses with individual mediators (Table 8): Socioemotional Adjustment, Cognitive-Scholastic Advantage, and Motivational Advantage each partially mediated the effect of CPC preschool on PW (33.3%, 27.8%, and 22.2% reductions, respectively). School and Community Support and Family Support Behavior were each associated with minimal reductions on the main effect of CPC preschool, indicating absent mediation (11.1% and 5.6% reductions, respectively).

In analyses with the full set of mediators (Table 9): Both the 5HM model and the 5HM-plus-adult-outcomes model fully mediated the effect of CPC preschool on PW (62.5% and 75.0% reductions, respectively).

##### No early ACEs.

In analyses with individual mediators (Table 8): Cognitive-Scholastic Advantage fully mediated the effect of CPC preschool on PW (75.0% reduction). Socioemotional Adjustment partially mediated the effect of CPC preschool on PW (37.5% reduction). Motivational Advantage was associated with minimal reductions on the main effect of CPC preschool (12.5% reduction). Family Support Behavior and School and Community Support did not reduce the effect of CPC preschool on PW.

In analyses with the full set of mediators (Table 9): Both the 5HM model and the 5HM-plus-adult-outcomes model fully mediated the effect of CPC preschool on PW (62.5% and 75.0% reductions, respectively).

##### Comparison across ACE groups.

In analyses with individual mediators (Table 8), CPC preschool made significantly greater contributions to the Cognitive-Scholastic Advantage (*d* = 0.25), Family Support Behavior (*d* = 0.23), and School and Community Support models (*d* = 0.21) among participants with ACE histories. In the full 5HM and 5HM-plus-adult-outcomes models, CPC preschool made similar contributions across ACE groups (*d* = 0.11 and 0.15, respectively; Table 9).

#### Early sociodemographic risk (Tables 8 and 9; Figure 5)

##### High early sociodemographic risk.

In analyses with individual mediators (Table 8): Cognitive-Scholastic Advantage and Socioemotional Adjustment fully mediated the effect of CPC preschool on PW (54.6% and 45.5% reductions, respectively). Family Support Behavior, Motivational Advantage, and School and Community Support were each associated with minimal reductions on the main effect of CPC preschool, indicating absent mediation (18.2%, 18.2%, and 9.1% reductions, respectively).

In analyses with the full set of mediators (Table 9): Both the 5HM model and the 5HM-plus-adult-outcomes model fully mediated the effect of CPC preschool on PW (63.6% and 81.8% reductions, respectively).

##### Low early sociodemographic risk.

In analyses with individual mediators (Table 8): Cognitive-Scholastic Advantage fully mediated the effect of CPC preschool on PW (50.0% reduction). Socioemotional Adjustment, Family Support Behavior, and School and Community Support were each associated with minimal reductions on the main effect of CPC preschool, indicating absent mediation (10.0% reductions each). Motivational Advantage did not reduce the effect of CPC preschool on PW.

In analyses with the full set of mediators (Table 9): the 5HM model and 5HM-plus-adult-outcomes models both partially mediated the effect of CPC preschool on PW (40.0% reductions each).

##### Comparison across sociodemographic risk groups.

CPC preschool made similar contributions across groups in analyses with individual mediators (*d* = 0.00–0.04; Table 8), the full 5HM model (*d* = 0.04; Table 9) and the 5HM-plus-adult-outcomes model (*d* = 0.07; Table 9).

## Discussion

To our knowledge, this is the first study to evaluate the association between participation in a public ECE intervention and long-term PW. Overall findings linked CPC participation to higher PW in early midlife (relative to a comparison group), with some differences in mediational across subgroups. The findings related to each research question will now be discussed in depth.

### Part I: Investigation of linkages between early childhood education and PW

Question 1: “Is CPC program participation (beginning in preschool) associated with better PW into early midlife (relative to a matched comparison group)?”

The present study’s findings supported the hypothesis that CPC participation would be linked to higher PW in adulthood. In the full sample, CPC preschool participants endorsed significantly higher PW at age 35–37 than comparison group members.

The CPC program was intentionally designed to promote children’s overall well-being. Indeed, one of the program’s original architects wrote that its aims were to “reach the child and parent early, develop language skills and self-confidence, and to demonstrate that these children, if given a chance, can meet successfully all the demands of today’s technological, urban society” (Sullivan, 1971). These aims align closely with Ryff and Keyes’ (1995) definition of PW as a multidimensional construct which broadly “includes positive evaluations of oneself and one’s past life … a sense of continued growth and development as a person … the belief that one’s life is purposeful and meaningful … the capacity to manage effectively one’s life and surrounding world … and a sense of self-determination” (p. 720).

The latter definition of PW broadly describes individuals’ schemas about themselves and the world around them – as developed over time in the context of past experiences, personal and cultural values (Stein, 1995). Early childhood has been identified as a critical period for schema development, particularly schemas regarding the self (Cicchetti, 1991; Harter, 1998). Related work has also shown that both schemas and PW (as measured by the RSPWB and similar measures) are relatively stable over time (Springer et al., 2011). These findings, coupled with the present study’s results, highlight the importance of promoting PW starting early in life.

The CPC program likely contributed to the latter goal by providing participants with stable, nurturing early learning environments, and by initiating positive developmental cascades. CPC participants were immersed in nurturing environments which aimed to promote not only academic learning, but holistic development (Sullivan, 1971). The CPC program also included a strong parent involvement component, which aimed to increase family members’ engagement in the educational process both at school and at home. These combined experiences surely sent strong positive messages to participants about their value as individuals, their ability to overcome challenges, and their potential to succeed. CPC participation has also been shown to initiate positive developmental cascades over time (e.g., increased academic achievement and admission to magnet schools), which likely contribute to long-term PW (e.g., Reynolds et al., 2007; Reynolds & Ou, 2011). These and other potential mechanisms linking CPC program participation to later PW will be discussed in more depth under Question 4.

Question 2: “Does the relationship between CPC program participation and early midlife PW differ for key subgroups (based on sex, early ACE history, and early family sociodemographic risk)?”

#### Sex

The hypothesis that CPC intervention would be associated with greater PW for males than females was not supported. Regression analyses controlling for salient demographic factors indicated that CPC preschool was associated with higher PW at age 35–37 for both sexes; however, its contributions to the model were similar across sexes. CPC follow-on intervention was not significantly related to PW for either sex. Overall, these results suggest that CPC preschool is associated with psychological benefits for both males and females. This heartening finding stands in contrast to previous research which found greater benefits of CPC participation for males in other domains (e.g., educational attainment; Ou & Reynolds, 2010; Reynolds et al., 2011). The discussion of Question 4 below will outline possible differences in the mechanisms that link CPC preschool to PW in males versus females.

#### Early ACEs

The hypothesis that CPC intervention would be associated with greater PW for individuals with early ACE histories (ages 0 to 5 years) was partially supported. Any CPC preschool participation was associated with higher PW in adulthood for both the any-ACE and no-ACE groups, with stronger effects in the any-ACE group. However, CPC follow-on participation was associated with lower PW for any-ACE participants only.

Taken together, these findings paint a complicated picture of the effects of early intervention on the PW of children with histories of early ACEs. On one hand, these findings highlight preschool as a critical intervention period for young children, especially those who have experienced maltreatment and other ACEs. In the present sample, ACE-affected children – many of whom had experienced significant instability and home and/or had limited access to early learning opportunities before prekindergarten – clearly benefitted from the stable, nurturing environment of the CPC preschool program. As previously noted, CPC preschool has also been shown to prevent additional maltreatment during the elementary school period, which undoubtedly impacted this group’s development (Mersky et al., 2011).

The linkage between CPC follow-on and lower PW in the any-ACE group is surprising and merits additional investigation. Future studies should carefully investigate the issue of dosage, including granular comparison of the baseline and attrition characteristics of different dosage groups. Such research may help to inform intervention tailoring, to ensure that the socio-emotional gains that children make during preschool are sustained over the long-term. To this end, it would also be valuable to closely examine and compare the socioemotional learning trajectories of children with and without ACEs. For example, past work has shown that young children with ACE histories may be more likely to experience behavioral control problems and conflict in ECE settings than children without ACE histories (Lipscomb et ah, 2021). These difficulties, if unaddressed, could have negative effects on children’s learning, relationships, and psychological well-being over time.

#### Early sociodemographic risk

The hypothesis that CPC intervention would be associated with greater PW for individuals with higher levels of early sociodemographic risk (ages zero to three) was partially supported. In the high early sociodemographic risk group, CPC preschool was associated with significantly higher PW; however, CPC follow-on was not significantly related to PW. Meanwhile, neither CPC preschool nor follow-on was associated with PW in the lower risk group.

Taken together, these results indicate that, in a sample of children who all attended low-income school districts, early intervention exerted the greatest impacts on long-term PW for those who entered school with the highest cumulative sociodemographic risk. These findings are consistent with past research indicating that children from the most disadvantaged backgrounds tend to exhibit the greatest gains following early intervention (Arteaga et al., 2014; Currie, 2001; Ou & Reynolds, 2010; Reynolds et al., 2011). For children with high levels of sociodemographic risk, the CPC preschool program’s nurturing, stable environment, attention to holistic child development, and emphasis on parent and family involvement all likely laid the foundations for long-term psychological well-being.

### Part II: Investigation of mechanisms

Question 3: “Does socio-emotional skill enhancement mediate the relationship between CPC participation and long-term PW?”

Question 3 focused on the connection between CPC participation and later PW. A priori hypotheses posited that Socioemotional Adjustment would mediate the relationship between CPC participation and PW. It was also posited that other 5HM mediators – Cognitive-Scholastic Advantage, Family Support Behavior, School and Community Support, and Motivational Advantage – would also mediate the latter relationship, but to a lesser degree than Socioemotional Adjustment (Figure 1).

The hypotheses above were partially supported in individual mediator models controlling for sociodemographic factors with the full sample (Figure 2). Socioemotional Adjustment mediators significantly reduced the effect of CPC preschool on PW in the full sample (36.4%); however, School and Community Support mediators were associated with the greatest main effect reductions (100.0%). This was followed by Cognitive-Scholastic Advantage (54.6%) and Socioemotional Adjustment (36.4%). Motivational Advantage and Family Support Behavior were associated with minimal reductions (18.2% and 9.1%, respectively). The full 5HM model reduced the effect of CPC on PW by 63.6%; the 5HM model plus adult outcomes (educational attainment and felony arrest) reduced the effect by 54.6%.

Broadly, these results suggest that CPC participation may have impacted long-term PW by initiating positive developmental cascades – particularly in the academic domain (as measured by School and Community Support). CPC participation increased children’s access to stable, nurturing educational environments in early childhood and beyond (e.g., by facilitating attendance at magnet middle schools), which in turn likely had impacts on many of the other 5HM mediators (e.g., academic achievement, as measured by Cognitive-Scholastic Advantage; emotional and behavioral functioning in the classroom, as measured by Socioemotional Adjustment). The complex interplay between School and Community Support and other 5HM mediators may partially account for its influence in the full sample. Question 4 below provides further insight into the relative contributions of each 5HM mediator for different subgroups.

Question 4: “Do mediational pathways from CPC program to early midlife PW vary by participant subgroup?”

#### By sex (Figure 3)

The hypothesis that Socioemotional Adjustment would more strongly mediate the relations between CPC participation and long-term PW for males was partially supported in sex-split models. Among females, Socioemotional Adjustment was the individual 5HM mediator associated with the greatest percent reductions in the effect of CPC preschool on PW. Among males, other 5HM mediators were associated with the greatest percent reductions in the effect of CPC preschool on PW; however, the percent reduction associated with Socioemotional Adjustment was greater among males than females. These findings paint a complicated picture of the various contributions to PW across the sexes in this sample.

More specifically, among females, Socioemotional Adjustment mediators were associated with the greatest reductions in the effect of CPC preschool on PW (44.4%), followed by School and Community Support and Motivational Advantage (both 22.2%). Cognitive-Scholastic Advantage and Family Support Behavior were associated with minimal reductions in the main effect (both 11.1%). The full 5HM model reduced the effect of CPC on PW by 66.7%; the 5HM model plus adult outcomes (educational attainment and felony arrest) reduced the effect by 77.8%. These results indicate that while Socioemotional Adjustment made the greatest contributions of any 5HM mediator, the comprehensive set of mediators plus adult outcomes accounted for the greatest portion of the effect of CPC on long-term PW for females. This is consistent with previous CLS research, which has found that the full set of 5HM mediators generally accounts for the most variance in multidimensional outcomes in the full sample (e.g., Reynolds & Ou, 2011).

Meanwhile, among males, Cognitive-Scholastic Advantage mediators were associated with the greatest reductions in the effect of CPC preschool on PW (91.7%), followed by Socioemotional Adjustment (66.7%); and Family Support Behavior, School and Community Support, and Motivational Advantage (each 41.7%). The full 5HM model reduced the effect of CPC on well-being by 91.7%; the 5HM model plus adult outcomes (educational attainment and felony arrest) reduced the effect by 100%. These results suggest that Cognitive-Scholastic Advantage may be the primary mechanism through which CPC participation affects males’ long-term PW, above and beyond other individual mediators. This result is particularly salient in light of other research highlighting persistent disparities in educational opportunities and academic achievement among African American boys (e.g., Davis, 2003; Wood et al., 2007). According to 5HM theory, males who participate in CPC preschool exhibit improved cognitive-scholastic abilities upon school entry, which in turn initiates positive trajectories of academic performance (e.g., better grades, reduced rates of grade retention). The latter experiences, in turn, may promote males’ lifelong sense of self-efficacy, motivation, and overall well-being (McCarty, 2008; Schweinhart et al., 1993).

Cognitive-Scholastic Advantage accounted for 91.7% of the effect of CPC preschool on PW among males; adding the other 5HM mediators did not change the percent reduction. However, adding the other 5HM mediators plus two adult outcomes (history of felony arrest and educational attainment by age 27) accounted for 100% of the effect of CPC preschool on PW. These results provide further evidence that Cognitive-Scholastic Advantage is the primary mechanism by which CPC preschool affects long-term PW for males, with additional modest contributions from adult outcomes. These results suggest that educational attainment alone is not the primary driver of males’ long-term PW. Rather, the skills and resources related to Cognitive-Scholastic Advantage (e.g., core math and reading skills, the ability to self-regulate and complete tasks) appear to be the primary contributors to males’ PW in early midlife. These skills and resources likely had significant influences on males’ abilities to navigate the complexities of the world around them – a world in which they, as men of color, face significant inequities.

#### By early ACE status (Figure 4)

The hypothesis that Socioemotional Adjustment would more strongly mediate the relations between CPC participation and long-term PW for individuals with early ACE histories was not supported in ACE history-split models. Among the any-ACE group, Socioemotional Adjustment was the individual 5HM mediator associated with the greatest percent reductions in the effect of CPC on PW; however, it was associated with slightly higher percent reductions in the no-ACE group. For both groups, the full set of 5HM mediators plus adult outcomes were associated with the greatest percent reductions in the effect of CPC on PW.

More specifically, among the any-ACE group, Socioemotional Adjustment mediators were associated with the greatest reductions in the effect of CPC preschool on PW (33.3%), followed by Cognitive-Scholastic Advantage (27.8%), and Motivational Advantage (22.2%). School and Community Support and Family Support Behavior were associated with minimal reductions in the main effect (11.1% and 5.6%, respectively). The full 5HM model reduced the effect of CPC on PW by 62.5%; the 5HM model plus adult outcomes (educational attainment and felony arrest) reduced the effect by 75.0%. These results indicate that while Socioemotional Adjustment made the greatest contributions of any 5HM mediator, the comprehensive set of mediators plus adult outcomes accounted for the greatest portion of the effect of CPC on long-term PW for participants with early ACE histories. The relative contributions of Socioemotional Adjustment align with previous research demonstrating that ACE-affected individuals are at increased risk for short- and long-term social, emotional, and psychological problems (e.g., Choi et al., 2017; Edwards et al., 2003; Giovanelli et al., 2016). Preschool intervention, in turn, may increase children’s social and emotional skills (e.g., abilities to build meaningful relationships with others, understand and regulate emotions, manage behavior) through the full array of 5HM mediators, laying a healthy foundation for lifelong PW (Reynolds & Ou, 2011).

Meanwhile, among the no-ACE group, Cognitive-Scholastic Advantage was associated with the greatest reductions in the effect of CPC preschool on PW (75.0%), followed by Socioemotional Adjustment (37.5%) and Motivational Advantage (12.5%). The full 5HM model reduced the effect of CPC on PW by 62.5%; the 5HM model plus adult outcomes (educational attainment and felony arrest) reduced the effect by 75.0%. These results indicate that the comprehensive set of mediators plus adult outcomes accounted for the greatest portion of the effect of CPC on long-term PW for the no-ACE group (e.g., participants who were low-income but did not have a history of adverse or traumatic events in early childhood). As noted for other findings, this is in keeping with previous CLS research, which has found that the full set of 5HM mediators generally accounts for the majority of outcome variance in the full sample (e.g., Reynolds & Ou, 2011).

#### By early sociodemographic risk (Figure 5)

The hypothesis that Socioemotional Adjustment would more strongly mediate the relations between CPC participation and long-term PW for individuals with higher levels of early sociodemographic risk was supported. Socioemotional Adjustment was associated with substantively higher percent reductions in the effect of CPC on PW among the higher risk group. However, other 5HM mediators also made significant contributions in the higher risk group.

More specifically, among the higher risk group, Cognitive-Scholastic Advantage mediators were associated with the greatest reductions in the effect of CPC preschool on PW (54.6%), followed by Socioemotional Adjustment (45.5%). Family Support Behavior, Motivational Advantage, and School and Community Support were associated with minimal reductions in the main effect (18.2%, 18.2%, and 9.1%). The full 5HM model reduced the effect of CPC on well-being by 63.6%; the 5HM model plus adult outcomes (educational attainment and felony arrest) reduced the effect by 81.8%. These findings indicate that the full 5HM model plus adult outcomes accounted for the greatest proportion of the effect of CPC preschool on PW. This likely reflects the complex impacts of deep poverty on all aspects of children’s development – cognitive, socio-emotional, physical, and more (Blair & Raver, 2012; Bolger et al., 1995; Dashiff et al., 2009; Nikulina et al., 2011; Saulsberry et al., 2013). CPC preschool, in turn, enhances children’s developmental trajectories by supporting early academic and socio-emotional learning, enhancing family and school support networks, facilitating access to social services, and increasing children’s intrinsic motivation and self-esteem. These combined factors – rather than any one set of mediators – may improve the long-term PW of individuals from the most disadvantaged backgrounds.

Members of the lower risk group also grew up in low-income school districts but were affected by relatively fewer family/socio-demographic risk factors. In this group, Cognitive-Scholastic Advantage mediators were associated with the greatest reductions in the effect of CPC preschool on PW (50.0%). Socioemotional Adjustment, Family Support Behavior, and School and Community Support were associated with minimal reductions in the main effect (each 10.0%). The full 5HM model reduced the effect of CPC on PW by 40.0%; adding adult outcomes to the 5HM model did not change the percent reduction (40.0%). This result indicates that, for individuals affected by less complex arrays of early risk, Cognitive-Scholastic Advantage was the primary mechanism via which CPC preschool influenced long-term PW. As was the case with males, these effects appear to be driven by increased cognitive skills and resources (e.g., core math and reading abilities, the ability to self-regulate and complete tasks) rather than educational attainment. These skills and resources enable individuals to effectively navigate the world and to make plans for their futures – both critical components of PW.

#### Summary across subgroups

Overall, the previous results indicate that, for males, participants with no early ACEs, and participants with relatively lower levels of early sociodemographic risk, Cognitive-Scholastic Advantage was the strongest mechanism of influence linking CPC participation and PW. Finally, for females, participants with early ACE histories and participants affected by high levels of early sociodemographic risk, the full 5HM model was associated with the greatest percent reductions of CPC participation on PW. As discussed above, these results suggest unique risk and protective processes leading to long-term PW for different subgroups. They also suggest that the relative importance of each 5HM mechanism likely differs across subgroups (Figure 1).

The Age 35–37 Survey is the first time that the CLS has measured PW using the RSPWB. However, the previously described 5HM results can be broadly compared to previous CLS mediation studies examining related outcomes. In an early study, Reynolds et al. (2004) investigated the 5HM mechanisms leading to high school completion and juvenile arrest. They reported that Cognitive Advantage, Family Support, and School Support were the strongest mechanisms of influence for these outcomes. Later, Reynolds and Ou (2011) examined the relations between 5HM variables and depressive symptoms at that time-point within an SEM framework. They reported that the full 5HM model was the best fit for the data, accounting for 79% of the main effect of CPC participation on symptomology. Cognitive-Scholastic Advantage (via increased parent involvement, higher rates of magnet school attendance, and lower rates of retention and special education placement), Socioemotional Adjustment (via early classroom adjustment), and Motivational Advantage (via school commitment) were the major paths through which CPC participation influenced depressive symptoms; School and Family Support made smaller contributions. Reynolds and Ou also applied the 5HM to other age 22–24 outcomes and found that Family and School Support directly mediated the effects of CPC preschool participation on educational outcomes and criminal justice system involvement.

Consistent with Reynolds and Ou’s (2011) findings on depressive symptoms, the present study found that the overall 5HM model, Cognitive-Scholastic Advantage, and Socioemotional Adjustment were associated with the greatest reductions in the main effect of CPC preschool on PW, depending on the subgroup. Motivational Advantage was less strongly related to PW at age 35–37 than it was to depressive symptoms at age 22–24. This is somewhat surprising, given that Motivational Advantage measured participants’ school commitment and expectations for their futures when they were children. Incorporating measures of motivation in late adolescence and early adulthood may have increased the total contributions to PW.

Family Support Behavior and School and Community Support were also less strongly related to PW in the present study than they were to previously studied non-psychological outcomes (e.g., educational attainment, criminal justice system involvement in early adulthood; Reynolds & Ou, 2011). School and Community Support significantly reduced the effects of CPC preschool on PW in the full sample; however, when results were parsed out by subgroup, School and Community Support made minimal contributions. Whereas Family Support Behavior and School and Community Support underscore support in the broader social-ecological system, Cognitive-Scholastic Advantage and Socioemotional Adjustment capture individual skills and resources that enable individuals to navigate their environments and overcome adversity. As with Motivational Advantage, it is possible that incorporating alternative measures of Family and School and Community Support (e.g., school climate, parent-child relationship quality, relationships with neighbors) may have increased these mediators’ total contributions to PW.

## Strengths and limitations

### Strengths

The present study has a number of strengths. First, this study examined a large sample of people of color (~93% African American, ~7% Hispanic) who grew up in urban poverty. This is a population which has been severely understudied in psychological research, suffers from significant health disparities, and for whom preventive strategies need to be identified. The CLS’ large sample size allows for well-powered investigations of the effects of early intervention on long-term well-being in this group, and for investigation of potential differential effects across different subgroups.

Second, the longitudinal nature of the CLS (spanning over four decades) is a major strength. The wealth of data collected over time, coupled with the study’s high sample recovery rate, allowed for investigation of numerous factors influencing long-term PW. Furthermore, the present study’s application of IPW increases confidence in the representativeness of the study sample, and in the accuracy of the results. The IPW model implemented in the present study is the most comprehensive attrition correction that has ever been utilized in the CLS.

Third, the present study’s investigation of the potential mechanisms linking CPC participation to long-term PW lays a valuable foundation for future research. Identification of program mechanisms increases scientific knowledge of how child development unfolds in early intervention contexts and may also inform future tailoring efforts.

### Limitations

The present study also has several key limitations. First, the study sample grew up in urban Chicago in the 1980s, and as such, their life experiences and trajectories may not fully generalize to other groups, including children growing up today. Emerging findings from the Midwest Longitudinal Study have supported the CPC program’s benefits for modern-day children from diverse cultural and geographic backgrounds; however, data on long-term mental health will take time to obtain (Reynolds et al., 2021).

Second, inference in the present study may be somewhat limited given the quasi-experimental design (as compared to a well-executed randomized controlled trial with low attrition). It is also important to note that causal relations among variables cannot be conclusively determined within a percent reduction framework (as is the case with all modeling approaches to mediation). With that being said, confidence in the importance of the measured mediators is increased by the fact that many of the models analyzed accounted for the main effect of CPC and also contributed unique variance to program outcomes.

Third, while the 5HM model includes a wide range of theoretically informed variables, it still may not fully capture either the five hypotheses or other relevant contributors to participants’ development. For example, the set of Socioemotional Adjustment variables does not include information about childhood mental health diagnoses (e.g., depression); Family Support Behavior does not include information about extended family and neighborhood support systems; and School and Community Support does not account for all aspects of the social and community context, including discrimination and racism. It is also possible that variables that were not available in the CLS data set (e.g., parental psychopathology, early parent–child interaction patterns) influenced the pattern of outcomes over time.

## Future directions

### Research

The present study indicates several important directions for future research. First, there is a need for additional prospective studies that investigate the relationship between ECE intervention, mental health, and other dimensions of well-being. There is a particular need for studies that begin in early life and that follow ECE graduates into adulthood. There is also a particular need for methodologically rigorous studies of populations affected by poverty and racism. It is important that these studies be adequately powered and well-controlled. Many previous studies have had significant design limitations (e.g., small sample sizes, lack of random assignment) which have affected the reliability and generalizability of results.

Second, from a transactional-ecological perspective, it will be important for future studies to carefully measure and assess the interplay of variables at multiple levels of analysis (e.g., biological, social, cognitive) and at multiple social-ecological levels (e.g., family, school, neighborhood). It will also be valuable to assess multiple aspects of psychological functioning (e.g., subclinical levels of symptomology, psychopathology, PW) with clinically rigorous measures. A major limitation of many previous studies has been the use of brief symptom screening measures, which may not provide comprehensive or reliable pictures of participants’ mental health, especially among participant populations that have been traditionally underrepresented in the mental health literature. Careful measurement will help to elucidate the developmental pathways that lead to either psychopathology or PW.

Third, as the present paper has outlined, the developmental pathways leading to PW may vary for different populations, and many questions remain about for whom and under what circumstances ECE interventions yield maximum impact. As such, it will be important for future studies to compare results across subgroups, and to investigate the biological, psychological, cognitive, and social mechanisms that link ECE interventions to long-term outcomes. It would be particularly valuable to investigate additional adult outcomes which may mediate the relationship between CPC participation and psychological outcomes (e.g., parenthood, physical health outcomes). Research in these veins may inform efforts to tailor ECE interventions to the needs of different populations.

Fourth, it will be important for future work to more fully explore the relationship between program dosage and PW. Future work should explore nonlinear patterns, as well as threshold effects of program dosage, consistent with previous CLS studies of other outcomes (e.g., educational attainment; Arteaga et al., 2014; Ou & Reynolds, 2008; Reynolds et al., 2019; Reynolds et al., 2018). Future work should also build on the present study by examining differential dosage patterns across different subgroups.

Fifth, future research should build on these results by investigating the mediational paths leading to PW within a structural equation modeling (SEM) framework. The latter approach would enable more nuanced investigation of latent variables, measurement error, and reciprocal causal relationships among variables (Beran & Violato, 2010; Gunzler et al., 2013; Jeon, 2015).

Finally, as sample sizes and the number of study variables increase, it will be critical to adequately address issues related to attrition bias. This is especially important to consider in the context of study samples of participants who grew up in contexts of socioeconomic risk and adversity. In longitudinal follow-ups, participants who continue to be affected by these risk factors are at increased risk for psychopathology and are also more likely to be lost to attrition (e.g., due to high residential mobility, lower social connectedness, and other factors). Researchers should continue to implement best practices for maintaining study samples over time (Ou et al., 2019) and should carefully apply advanced statistical techniques to address missing data (Seaman & White, 2013).

### Intervention and public policy

Although effectiveness is surely the purpose of all children’s programs, exactly what are they effective in doing? Whether a program is worth the effort and money depends not just on whether it achieves its goals but also on how valuable the goals are to the participants and to society (Zigler, 1998, p. 6).

Most ECE programs were initially designed with the main goal of promoting school readiness and educational attainment. The latter outcomes are beneficial not only for individuals and their families but also for society at large. Yet at the individual level, academic achievement and educational attainment are ultimately meaningful in that they enable individuals to navigate the world around them, and to lead fulfilling, productive lives – even in the face of stress and adversity. This is the broad definition of psychological well-being.

Overall, the results of the present study indicate that CPC preschool participation may lay a strong foundation for long-term PW, but that program participation is not a total panacea. As commonly noted by Edward Zigler, one of the architects of the Head Start, early intervention is not an inoculation against adversity or negative outcomes for children growing up in poverty. A combination of interventions at multiple social-ecological levels (e.g., individual, family, school, community) and public policies aimed at promoting health and educational equity will likely be most effective in promoting lifelong well-being among individuals affected by poverty and other psychosocial risk factors.

## Figures and Tables

**Figure 4.**
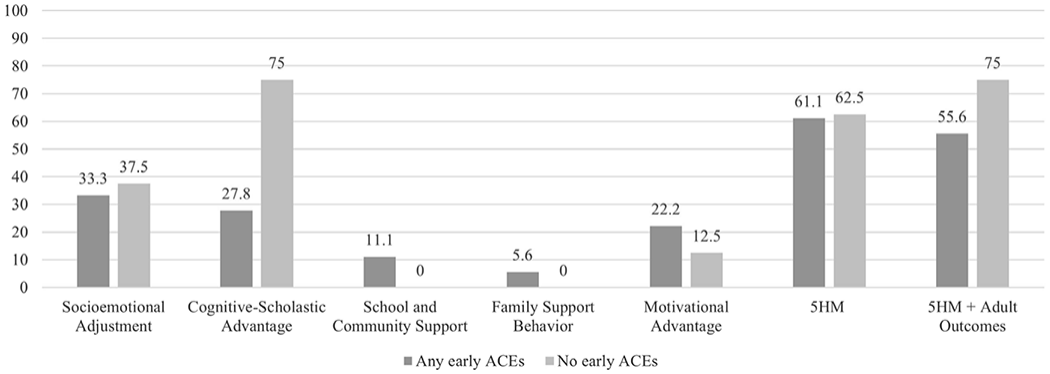
5HM mediators – Percent reductions for the effect of CPC preschool on overall PW – By early ACE status.

**Figure 5.**
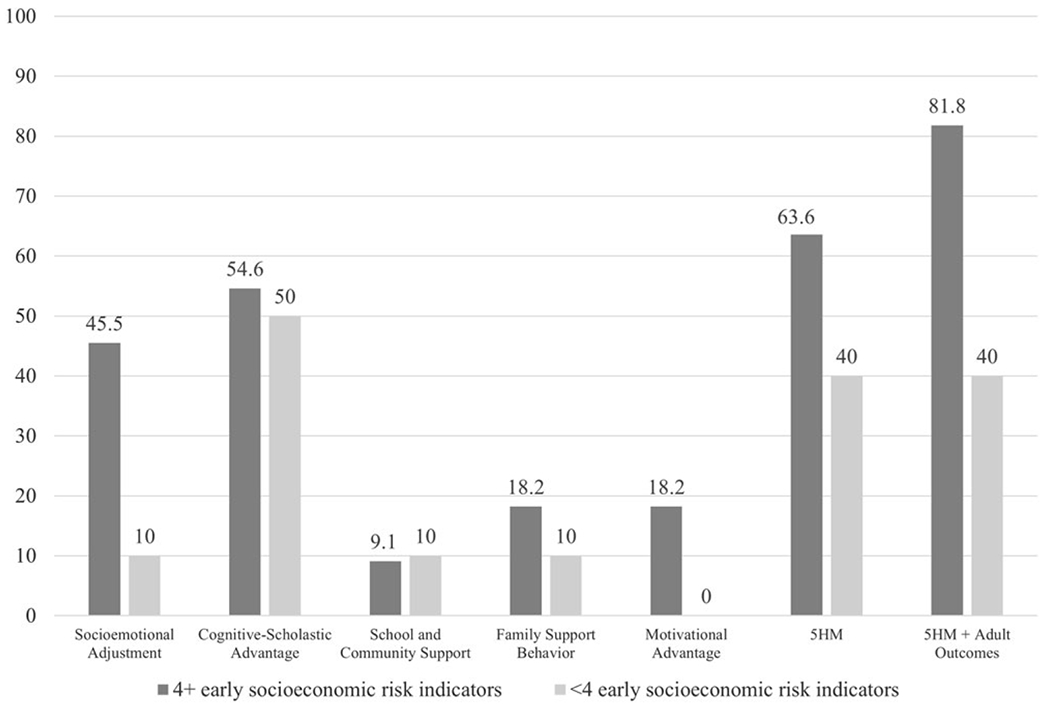
5HM mediators – Percent reductions for the effect of CPC preschool on overall PW – By early sociodemographic risk.

**Table 1.** Sample characteristics

	Original CLS sample (*N* = 1,531)	Age 35–37 survey sample (*N* = 1,104)	Living participants lost to attrition at age 35–37 (*N* = 371)	Age 35–37 survey sample with IPW (*N* = 1,104)
Female	50.4%	54.4%	42.9%	51.2%
Male	49.6%	45.6%	57.1%	48.8%
African American	93.0%	93.7%	90.8%	92.8%
Hispanic	7.0%	6.3%	9.2%	7.2%
CPC preschool	64.3%	65.8%	59.3%	64.2%
Extended CPC	35.9%	37.7%	31.5%	36.1%
Age 0–3 risk index (*mean*)	4.52 (*SD* = 1.69)	4.44 (*SD* = 1.68)	4.72 (*SD* = 1.70)	4.50 (*SD* = 1.69)
-Resided in a single parent household	76.5%	75.4%	79.0%	76.4%
-Mother under age 18 at child’s birth	16.2%	15.0%	20.2%	16.3%
- Four or more children in household	16.6%	16.9%	16.4%	16.9%
-Mother did not graduate high school	54.3%	52.4%	59.0%	53.7%
-Family income was below 185% of the federal poverty level	62.8%	60.9%	66.8%	61.9%
-Mother was unemployed or part-time	66.3%	65.4%	67.1%	65.4%
-Family received federal welfare	83.8%	83.0%	85.4%	83.3%
-High-poverty school attendance area	76.0%	75.3%	78.2%	75.8%
% of persons in poverty by census tract at time of birth (1980; *mean*)	42.3%	41.7%	43.4%	41.8%
Any history of juvenile arrest	30.6%	28.7%	33.4%	30.2%
Graduated from high school by August 2008 (age ~28)	55.8%	55.6%	32.6%	49.4%
On-time high school graduation	40.7%	44.7%	28.0%	38.9%

*Note.* The original CLS sample included 1,539 participants; 8 individuals were excluded because identifying information was unavailable.

* *p* < 0.05.

** *p* < 0.01.

*** *p* < 0.001.

**Table 2.** Predictor variables for age 35–37 sample retention in sex-split IPW model (*N* = 1,531–1,539)

Variable	Variable description	*Mean* (*SD*)	% of sample (dichotomous variables)
1	CPC preschool participation	–	64.30
2	School-age CPC participation	–	55.20
3	African American	–	93.00
4	Low birth weight	–	11.80
5	Word analysis skills at the end of kindergarten	63.82 (13.25)	–
6	Composite school readiness score	47.37 (8.77)	–
7	Substantiated maltreatment between ages 0 to 3 years	–	3.80
8	Mother was not a high school graduate by participant age three	–	54.30
9	Eligible for free lunch between ages zero and three	–	83.80
10	Mother was under age 18 at the participant’s birth	–	16.20
11	Lived in a household of four or more children between ages zero and three	–	16.60
12	Family income was below 185% of the federal poverty level between ages zero and three	–	62.80
13	Mother was unemployed or employed part-time when participant was between ages zero and three	–	66.30
14	Lived in a single parent household between ages zero and three	–	76.50
15	Information was not available about age zero to three risk indicators (items 9–15 above)	–	16.20
16	Lived in a school attendance area where at least 60% of households were impoverished	–	76.00
17	Interaction term: CPC preschool * age zero to three sociodemographic risk index	2.90 (2.54)	–
18	% individuals living 1 year within the participants’ housing unit by age four	0.19 (0.07)	–
19	% individuals living between 1 and 5 years within the participants’ housing unit by age four	0.29 (0.07)	–
20	% individuals living 5 to 10 years within the participants’ housing unit by age four	0.23 (0.09)	–
21	% individuals living 10 to 20 years within the participants’ housing unit by age four	0.25 (0.11)	–
22	% self-employed individuals ages 16 and older within the participant’s census tract by age four	0.02 (0.02)	–
23	% female-headed black households within the participant’s census tract by age four	0.40 (0.15)	–
24	Frequent family conflict between ages zero and five	–	5.70
25	Family financial problems between ages zero and five	–	7.00
26	Parental substance abuse problems between ages zero and five	–	4.10
27	Socio-demographic risk index (age eight)	4.25 (1.79)	–
28	Socio-demographic risk index (age 12)	4.23 (1.82)	–
29	Grades 1–3 teacher-rated socioemotional functioning	19.14 (4.67)	–
30	Grades 4–6 teacher-rated socioemotional functioning	18.54 (4.45)	–
31	% birth tract population, ages 25+ years, with 4 or more years of college attendance in 1990	5.75 (6.47)	–
32	Number of years of magnet school attendance between fourth and eighth grade	0.40 (1.29)	–
33	Eighth grade reading score	145.0 (20.74)	–
34	Number of years active in Chicago Public Schools between kindergarten and 12th grade	9.04 (3.54)	–
35	Number of school moves between kindergarten and 12th grade	3.04 (1.50)	–
36	Dropped out of high school before age 16	–	12.5
37	Graduated from high school on-time	–	37.8
38	Socio-demographic risk index (age 17)	3.74 (1.48)	–
39	Ever arrested before age 18	–	30.6
40	Number of felony arrests by age 26	0.48 (1.15)	–
41	Socioeconomic Status Index (ages 24–27) – includes average annual earnings from 2004–2007 and educational attainment by age 26.	2.99 (2.11)	–
42	“Stable employment” (ages 24–27) – 8 or more quarters with any earnings from 2004–2007.	–	45.8
43	Average annual earnings (ages 24–27).	1.13 (1.30)	–
44	Moderate or higher occupational prestige/skill (age 24–27).	–	23.9
45	Highest grade completed by August 2008	12.06 (1.65)	–
46	Number of missing adult outcomes (items 41–46)	4.17 (2.38)	–
47	CLS located a Social Security Number by 2007	–	93.5
48	Number of years received TANF (ages 24–27).	0.10 (0.40)	–
49	Last known address as of July 2017 was in Illinois	–	76.9

**Table 3.** Bivariate correlations among Ryff scales of psychological well-being items in the unweighted, full sample

Item	1.	2.	3.	4.	5–18.
1. When I look at the story of my life, I am pleased with how things have turned out so far.	–	–	–	–	–
2. Some people wander aimlessly through life but I am not one of them.	0.32***	–	–	–	–
3. The demands of everyday life often get me down.	−0.27***	–0.14***	–	–	–
4. In many ways, I feel disappointed about my achievements in life.	−0.50***	−0.25***	0.40***	–	
5. Maintaining close relationships has been difficult and frustrating for me.	−0.30***	−0.19***	0.39***	0.40***	

Item	1.	2.	3.	4.	5.	6.	7.	8–18.
6. In general, I feel I am in charge of the situation in which I live.	0.34***	0.18***	−0.20***	−0.21***	−0.22***	–	–	
7. I am good at managing the responsibilities of daily life.	0.29***	0.27***	−0.24***	−0.24***	−0.23***	0.37***	–	
8. I have not experienced many warm and trusting relationships with others.	−0.25***	−0.16***	0.27***	0.35***	0.46***	−0.19***	−0.19***	–

Item	1.	2.	3.	4.	5.	6.	7.	8.	9.	10.	11–18.
9. I live life 1 day at a time and do not really think about the future.	−0.08	−0.08**	0.16***	0.16***	0.16***	−0.01	−0.13 ***	0.22***	–	–	–
10. I sometimes feel as if I have done all there is to do in life.	0.02	−0.05	0.14***	0.14***	0.13***	−0.05	−0.06*	0.22***	0.27***	–	–
11. I gave up trying to make big improvements or changes in my life a long time ago.	−0.06	−0.06*	0.20***	0.20***	0.23***	−0.08 **	−0.15 ***	0.28***	0.27***	0.33***	–

Item	1.	2.	3.	4.	5.	6.	7.	8.	9.	10.	11.	12.	13–18.
12. I tend to be influenced by people with strong opinions.	−0.08**	−0.06	0.21***	0.18 ***	0.19***	−0.07*	−0.07*	0.21***	0.20***	0.20***	0.27***	–	

Item	1.	2.	3.	4.	5.	6.	7.	8.	9.	10.	11.	12.	13–18.
13. For me, life has been a continuous process of learning, changing, and growth.	0.09 **	0.11 ***	−0.09**	−0.09**	−0.07*	0.11***	0.20***	−0.07*	−0.11***	−0.08**	−0.11 ***	−0.08**	

Item	1.	2.	3.	4.	5.	6.	7.	8.	9.	10.	11.	12.	13.	14–18.
14. I think it is important to have new experiences that challenge how I think about myself and the world.	0.05	0.09**	−0.06*	−0.09**	−0.06	0.15***	0.20***	−0.04	−0.03	−0.10 ***	−0.10 ***	−0.03	−0.03	0.40***	–

Item	1.	2.	3.	4.	5.	6.	7.	8.	9.	10.	11.	12.	13.	14.	15–18.
15. I have confidence in my own opinions, even if they are different from the way most other people think.	0.08**	0.14***	−0.06 *	−0.11 ***	−0.07 *	0.11***	0.14***	0.00	−0.01	0.01	−0.07 *	−0.01	0.19***	0.23***	–

Item	1.	2.	3.	4.	5.	6.	7.	8.	9.	10.	11.	12.	13.	14.	15.	16.	17–18.
16. I judge myself by what I think is important, not by the values of what others think is important.	0.11 ***	0.14 ***	−0.10 ***	−0.08 **	−0.05	0.18 ***	0.14 ***	0.02	0.02	−0.02	0.01	−0.09 **	0.11 ***	0.13 ***	0.30 ***	–	–
17. I like most parts of my personality.	0.22 ***	0.23 ***	−0.13 ***	−0.14 ***	−0.20 ***	0.20 ***	0.28 ***	0.22 ***	−0.01	0.01	−0.06*	0.00	0.11 ***	0.16 ***	0.18 ***	0.11 ***	–

Item	1.	2.	3.	4.	5.	6.	7.	8.	9.	10.	11.	12.	13.	14.	15.	16.	17.	18.
18. People would describe me as a giving person, willing to share my time with others.	0.12 ***	0.14 ***	−0.01	−0.04	−0.09 **	0.11 ***	0.26 ***	−0.09 **	−0.05	0.00	−0.03	0.03	0.14 ***	0.14 ***	0.15 ***	0.10 ***	0.23 ***	–

* *p* < 0.05.

** *p* < 0.01.

*** *p* < 0.001.

**Table 4.** Correlations between PW and other key variables – unweighted, full sample

	Overall PW
CPC participation	
CPC preschool (dichotomous, 1 = yes)	0.10***
# years CPC preschool	0.09**
Any CPC follow-on	0.00
Extended CPC (4+ years)	0.06*
Adult outcomes	
Highest grade completed by 2015	0.21***
Average annual income, 2010–2014	0.23***
Ever jailed or incarcerated by 2015	−0.21***
Brief Symptom Inventory continuous depression score (age 35–37)	−0.48**

* *p* < 0.05.

** *p* < 0.01.

*** *p* < 0.001.

**Table 5.** Ryff scales of psychological well-being – descriptive statistics with and without IPW

	Overall PW – *Mean* (*SD*)	
Full sample		
*Mean* (*SD*) – Unweighted	92.79 (10.97)	
*Mean* (*SD*) – IPW	92.44 (11.01)	
CPC versus comparison		
CPC prek		
*Mean* (*SD*) – Unweighted	93.60 (10.45)	
*Mean* (*SD*) – IPW	93.17 (10.67)	
Comparison group		
*Mean* (*SD*) – Unweighted	91.24 (11.76)	
*Mean* (*SD*) – IPW	91.14 (11.74)	
CPC versus comparison individual samples *t*-test (with IPW)		*t* = −3.31, *df* = 1418, *p* < 0.00)
	Overall PW – *Mean* (*SD*)	
By sex		
Males		
*Mean* (*SD*) – Unweighted	91.69 (11.29)	
*Mean* (*SD*) – IPW	91.25 (11.36)	
Females		
*Mean* (*SD*) – Unweighted	93.77 (10.58)	
*Mean* (*SD*) – IPW	93.58 (10.74)	
Males versus females individual samples *t*-test (with IPW)		*t* = 3.97, *df* = 1418, *p* < 0.00
By early ACE status		
Any early ACEs		
*Mean* (*SD*) – Unweighted	90.63 (11.76)	
*Mean* (*SD*) – IPW	90.12 (12.01)	
By early ACE status (continued)	Overall PW – *Mean* (*SD*)	
No early ACEs		
*Mean* (*SD*) – Unweighted	93.37 (10.68)	
*Mean* (*SD*) – IPW	93.05 (10.77)	
Any versus no early ACEs individual samples *t*-test (with IPW)		*t* = 4.06, *df* = 1418, *p* < 0.00
By early socioeconomic risk status		
4+ early socioeconomic risk indicators		
*Mean* (*SD*) – Unweighted	92.13 (11.22)	
*Mean* (*SD*) – IPW	91.72 (11.34)	
By early socioeconomic risk status (continued)		
<4 early socioeconomic risk indicators		
*Mean* (*SD*) – Unweighted	94.57 (10.06)	
*Mean* (*SD*) – IPW	94.33 (10.23)	
4+ versus <4 early socioeconomic risk indicators individual samples *t*-test (with IPW)		*t* = 3.97, *df* = 1418, *p* < 0.00

**Table 6.** Regressions predicting PW at age 35, with IPW correction – full sample

	Total PW			
	*B*	*SE*	*β*	95% CI
Males	−2.24***	0.67	−0.10***	−3.56, −0.92
Black	0.46	1.31	0.01	−2.11, 3.02
Low birthweight	−0.87	1.03	−0.03	−2.90, 1.15
% birth census tract in poverty	−0.63	0.70	−0.03	−2.00, 0.74
CPC preschool	2.42**	0.78	0.11**	0.90, 3.94
CPC follow-on	−0.95	0.74	−0.04	−2.39, 0.50
ACEs ages 0–5	−1.26*	0.52	−0.07*	−2.29, −0.23
Sociodemographic risk ages 0–3	−0.76***	0.20	−0.11***	−1.15, −0.36

* *p* < 0.05.

** *p* < 0.01.

*** *p* < 0.001.

**Table 7.** Regressions predicting age 35 PW, with IPW correction – by sex, early ACEs, and early socioeconomic risk

	Males				Females				
	*B*	*SE*	*β*	95% CI	*B*	*SE*	*β*	95% CI	Cohen’s *d*
Males	–	–	–	–	–	–	–	–	–

Black	1.45	1.61	0.04	−2.34, 5.25	−0.85	1.61	−0.02	−4.40, 2.70	0.21

Low birthweight	−0.71	1.37	−0.02	−3.92, 2.51	−1.07	1.18	−0.03	−3.67, 1.53	0.03

% birth census tract in poverty	−1.09	0.89	−0.05	−3.19, 1.00	−0.14	0.83	−0.01	−1.96, 1.69	0.09

CPC preschool	2.69*	0.97	0.12*	0.42, 4.96	2.09*	0.95	0.09*	0.01, 4.17	0.05

CPC follow-on	−0.82	0.93	−0.04	−3.00, 1.37	−1.06	0.88	−0.05	−3.00, 0.88	0.02

ACEs ages 0–5	−1.12	−0.08	−0.08	−2.58, 0.21	−1.41	0.72	−0.07	−3.00, 0.17	0.03

Sociodemographic risk ages 0–3	−0.65*	0.26	−0.10*	−1.26, −0.04	−0.87***	0.24	−0.14***	−1.41, −0.34	0.02
	Any age 0–5 ACEs				No age 0–5 ACEs				
	*B*	*SE*	*β*	95% CI	*B*	*SE*	*B*	95% CI	Cohen’s *d*

Males	−2.33	1.37	−0.10	−5.01, 0.36	−2.26***	0.64	−0.11***	−3.71, −0.81	0.00

Black	3.56	3.21	0.06	−2.76, 9.88	0.13	1.20	0.00	–	0.31

Low birthweight	0.68	1.95	0.02	−3.15, 4.51	−1.28	1.00	−0.04	−3.54, 1.00	0.18

% birth census tract in poverty	−0.74	1.45	−0.03	−3.59, 2.10	−0.57	0.67	−0.03	−2.06, 0.94	0.02

CPC preschool	4.64**	1.56	0.18**	1.58, 7.70	1.74*	0.74	0.08*	0.05, 3.43	0.26

CPC follow-on	−4.63**	1.48	−0.19**	−7.54, −1.71	0.06	0.70	0.00	−1.53, 1.66	0.43

ACEs ages 0–5	–	–	–	–	–	–	–	–	–

Sociodemographic risk ages 0–3	−1.01*	0.42	−0.14*	−1.84, −0.19	−0.66**	0.19	−0.10**	−1.10, −0.22	0.03
	4+ early socioeconomic risk indicators				<4 early socioeconomic risk indicators				
	*B*	*SE*	*β*	95% CI	*B*	*SE*	*β*	95% CI	Cohen’s *d*

Males	−2.19**	0.71	−0.10**	−3.80, −0.59	−2.28*	1.04	−0.11*	−4.61, 0.05	0.01

Black	0.13	1.46	0.00	−3.21, 3.46	0.67	1.75	0.02	−3.27, 4.61	0.07

Low birthweight	−1.20	1.00	−0.04	−3.49, 1.09	1.34	2.09	0.03	−3.35, 6.03	0.23

% birth census tract in poverty	−0.71	0.72	−0.03	−2.35, 0.93	−1.07	1.11	−0.05	−3.56, 1.42	0.03

CPC preschool	2.52**	0.80	0.11**	0.69, 4.34	2.14	1.26	0.10	−0.70, 4.97	0.03

CPC follow-on	−1.35	0.76	−0.06	−3.08, 0.38	0.31	1.21	0.02	−2.40, 3.03	0.15

ACEs ages 0–5	−1.30*	0.56	−0.07*	−2.58, −0.03	−1.24	0.78	−0.08	2.99, 0.51	0.01

Sociodemographic risk ages 0–3	–	–	–	–	–	–	–	–	–

* *p* < 0.05.

** *p* < 0.01.

*** *p* < 0.001.

**Table 8.** Predicting overall PW – with demographic covariates and individual sets of 5HM mediators; with IPW correction

	Full sample			
Model	*B* for any CPC prek	*SE* for any CPC prek	*β* for any CPC prek	% reduction from Model 1
Model 1	2.42***	0.78	0.11***	–

Model 2	1.51*	0.68	0.07*	36.4%

Model 3	1.05	0.68	0.05	54.6%

Model 4	2.22**	0.69	0.10**	9.1%

Model 5	2.18	0.70	−0.05	100.0%

Model 6	2.07**	0.68	0.09**	18.2%

	Males				Females				
Model	*B* for any CPC prek	*SE* for any CPC prek	*β* for any CPC prek	% reduction from Model 1	*B* for any CPC prek	*SE* for any CPC prek	*β* for any CPC prek	% reduction from Model 1	Cohen’s *d*
Model 1	2.69*	0.97	0.12*	–	2.09*	0.95	0.09*	–	0.05

Model 2	0.89	0.95	0.04	66.7	1.13	0.94	0.05	44.4	0.02

Model 3	0.14	0.97	0.01	91.7	0.77	0.94	0.08	11.1	0.06

Model 4	1.65	0.97	0.07	41.7	1.74	0.95	0.08	11.1	0.01

Model 5	1.58	0.98	0.07	41.7	1.67	0.97	0.07	22.2	0.01

Model 6	1.51	0.96	0.07	41.7	1.57	0.94	0.07	22.2	0.01

	Any early ACEs				No early ACEs				
Model	*B* for any CPC prek	*SE* for any CPC prek	*β* for any CPC prek	% reduction from Model 1	*B* for any CPC prek	*SE* for any CPC prek	*β* for any CPC prek	% reduction from Model 1	Cohen’s *d*
Model 1	4.64**	1.56	0.18**	–	1.74*	0.74	0.08*	–	0.26

Model 2	3.04	1.76	0.12	33.3	1.03	0.85	0.05	37.5	0.18

Model 3	3.23	1.79	0.13	27.8	0.51	0.86	0.02	75.0	0.25

Model 4	4.26*	1.75	0.17*	5.6	1.71*	0.86	0.08*	–	0.23

Model 5	4.03*	1.78	0.16*	11.1	1.68	0.87	0.08	–	0.21

Model 6	3.45*	1.75	0.14*	22.2	1.56	0.85	0.07	12.5	0.17

	4+ early socioeconomic risk indicators				<4 early socioeconomic risk indicators				
Model	*B* for any CPC prek	*SE* for any CPC prek	*β* for any CPC prek	% reduction from Model 1	*B* for any CPC prek	*SE* for any CPC prek	*β* for any CPC prek	% reduction from Model 1	Cohen’s *d*
Model 1	2.52**	0.80	0.11**	–	2.14	1.26	0.10	–	0.03

Model 2	1.45	0.91	0.06	45.5	1.91	1.46	0.09	10.0	0.04

Model 3	1.07	0.92	0.05	54.6	1.05	1.47	0.05	50.0	0.00

Model 4	2.12*	0.92	0.09*	18.2	1.98	1.44	0.09	10.0	0.01

Model 5	2.29*	0.94	0.10*	9.1	1.90	1.45	0.09	10.0	0.04

Model 6	2.14*	0.92	0.09*	18.2	2.05	1.42	0.10	–	0.01

*Note.* Model 1 contains Block 1 only (Covariates: Males, Black, low birthweight, birth tract neighborhood poverty, any CPC prek, any CPC follow-on, age 0–5 ACEs, and age 0–3 sociodemographic risk). In Model 2, Block 2 = 5HM Socioemotional Adjustment (Covariates: teacher-rated socioemotional functioning (grades 1–3), frustration tolerance (grades 6–7), task orientation (grades 6–7), any juvenile arrest.) In Model 3, Block 2 = 5HM Cognitive-Scholastic Advantage. (Covariates: Kindergarten readiness score, retained or placed in special education (grades 1–8), 3rd grade reading score, 8th grade reading score.) In Model 4, Block 2 = 5HM Family Support Behavior. (Covariates: child maltreatment (ages 4–17), parent expectations of educational attainment, teacher-rated parent involvement (average of grades 1–3).) In Model 5, Block 2 = 5HM School and Community Support. (Covariates: = magnet school attendance (grades 4–8), moved schools (grades 4–8).) In Model 6, Block 2 = 5HM Motivational Advantage. (Covariates: commitment, expectations for educational attainment. Adult outcomes: Number of felony arrests by age 26, highest grade completed by age 27.)

* *p* < 0.05.

** *p* < 0.01.

*** *p* < 0.001.

**Table 9.** Predicting overall PW – with demographic covariates, 5HM, and adult outcomes; with IPW correction

	Full sample				Males				Females				
Model	*B* for any CPC prek	*SE* for any CPC prek	*β* for any CPC prek	% reduction from Model 1	*β* for any CPC prek	*SE* for any CPC prek	*β* for any CPC prek	% reduction from Model 1	*B* for any CPC prek	*SE* for any CPC prek	*β* for any CPC prek	% reduction from Model 1	Cohen’s *d*
Model 1 (Block 1 only)	2.42***	0.78	0.11***	–	2.69*	0.97	0.12*	–	2.09*	0.95	0.09*	–	0.05

Model 2 (Blocks 2–6: 5HM)	1.05	0.78	0.05	54.6	0.30	0.97	0.01	91.7	0.71	0.94	0.03	66.7	0.04

Model 3 (Blocks 2–6: 5HM; Block 7: Adult outcomes)	0.85	0.77	0.04	63.6	−0.90	1.02	−0.04	100.0	0.43	0.97	0.02	77.8	0.12

	Any early ACEs				No early ACEs				
Model	*B* for any CPC prek	*SE* for any CPC prek	*β* for any CPC prek	% reduction from Model 1	*B* for any CPC prek	*SE* for any CPC prek	*β* for any CPC prek	% reduction from Model 1	Cohen’s *d*
Model 1 (Block 1 only)	4.64**	1.56	0.18**	–	1.74*	0.74	0.08*	–	0.26

Model 2 (Blocks 2–6: 5HM)	1.87	1.78	0.07	61.1	0.70	0.86	0.03	62.5	0.11

Model 3 (Blocks 2–6: 5HM; Block 7: Adult outcomes)	2.01	1.78	0.08	55.6	0.40	0.86	0.02	75.0	0.15

	4+ early socioeconomic risk indicators				<4 early socioeconomic risk indicators				
Model	*B* for any CPC prek	*SE* for any CPC prek	*β* for any CPC prek	% reduction from Model 1	*B* for any CPC prek	*SE* for any CPC prek	*β* for any CPC prek	% reduction from Model 1	Cohen’s *d*
Model 1 (Block 1 only)	2.52**	0.80	0.11**	–	2.14	1.26	0.10	–	0.03

Model 2 (Blocks 2–6: 5HM)	0.83	0.93	0.04	63.6	1.24	1.48	0.06	40.0	0.04

Model 3 (Blocks 2–6: 5HM; Block 7: Adult outcomes)	0.55	0.92	0.02	81.8	1.28	1.46	0.06	40.0	0.07

*Note.* Model 1 contains Block 1 only (covariates: Males, Black, low birthweight, birth tract neighborhood poverty, any CPC prek, any CPC follow-on, age 0–5 ACEs, and age 0–3 sociodemographic risk). Model 2 contains each 5HM component: Socioemotional Adjustment (covariates: teacher-rated socioemotional functioning (grades 1–3), frustration tolerance (grades 6–7), task orientation (grades 6–7), any juvenile arrest), Cognitive-Scholastic Advantage (covariates: Kindergarten readiness score, retained or placed in special education (grades 1–8), 3rd grade reading score, 8th grade reading score), Family Support Behavior (covariates: child maltreatment (ages 4–17), parent expectations of educational attainment, teacher-rated parent involvement (average of grades 1–3)), School and Community Support (covariates: = magnet school attendance (grades 4–8), moved schools (grades 4–8)) and Motivational Advantage (covariates: commitment, expectations for educational attainment. Model 7 includes adult outcomes (covariates: number of felony arrests by age 26, highest grade completed by age 27).

* *p* < 0.05.

** *p* < 0.01.

*** *p* < 0.001.
